# Escaping Deleterious Immune Response in Their Hosts: Lessons from Trypanosomatids

**DOI:** 10.3389/fimmu.2016.00212

**Published:** 2016-05-31

**Authors:** Anne Geiger, Géraldine Bossard, Denis Sereno, Joana Pissarra, Jean-Loup Lemesre, Philippe Vincendeau, Philippe Holzmuller

**Affiliations:** ^1^UMR INTERTRYP, IRD-CIRAD, CIRAD TA A-17/G, Montpellier, France; ^2^UMR 177, IRD-CIRAD Université de Bordeaux Laboratoire de Parasitologie, Bordeaux, France; ^3^UMRCMAEE CIRAD-INRA TA-A15/G “Contrôle des maladies animales exotiques et émergentes”, Montpellier, France

**Keywords:** Trypanosomatidae family, parasite–host interactions, immunosuppression, *Leishmania* sp., *Trypanosoma brucei* sp., *Trypanosoma cruzi*

## Abstract

The Trypanosomatidae family includes the genera *Trypanosoma* and *Leishmania*, protozoan parasites displaying complex digenetic life cycles requiring a vertebrate host and an insect vector. *Trypanosoma brucei gambiense*, *Trypanosoma cruzi*, and *Leishmania* spp. are important human pathogens causing human African trypanosomiasis (HAT or sleeping sickness), Chagas’ disease, and various clinical forms of Leishmaniasis, respectively. They are transmitted to humans by tsetse flies, triatomine bugs, or sandflies, and affect millions of people worldwide. In humans, extracellular African trypanosomes (*T. brucei*) evade the hosts’ immune defenses, allowing their transmission to the next host, via the tsetse vector. By contrast, *T. cruzi* and *Leishmania* sp. have developed a complex intracellular lifestyle, also preventing several mechanisms to circumvent the host’s immune response. This review seeks to set out the immune evasion strategies developed by the different trypanosomatids resulting from parasite–host interactions and will focus on: clinical and epidemiological importance of diseases; life cycles: parasites–hosts–vectors; innate immunity: key steps for trypanosomatids in invading hosts; deregulation of antigen-presenting cells; disruption of efficient specific immunity; and the immune responses used for parasite proliferation.

## Clinical and Epidemiological Importance of Neglected Diseases

Trypanosomatid parasites interact with a wide range of insects and mammals to complete their life cycles. Some species, particularly *Trypanosoma brucei gambiense, Trypanosoma brucei rhodesiense*, *Trypanosoma cruzi*, and *Leishmania* spp. are pathogenic for humans, causing, respectively, human African trypanosomiasis (HAT or sleeping sickness), Chagas’ disease, and cutaneous, mucocutaneaous, and visceral Leishmaniasis (VL). These infectious eukaryotic parasites have been described and identified over a century ago; however, as of today, no vaccines are available and the availability of effective prophylactic and therapeutic drugs remains limited. It is estimated that more than 20 million people are infected and that 100,000 people die each year of trypanosomiasis or Chagas’ disease (1). Annually, cutaneous leishmaniasis affects around 1 million people, whereas VL is responsible for around 500,000 cases annually resulting in over 50,000 deaths (2).

Tsetse flies (*Glossina* spp.) transmit HAT-causing trypanosomes. Regarding mortality, it ranks 9th out of 25 human infectious and parasitic diseases in Africa (3) and is estimated to cause the loss of 1.5 million disability-adjusted life years per year (4). It is responsible for major setbacks in social, agricultural, and economic development in Africa (5) and constitutes a severe burden for poor rural populations to whom healthcare access is extremely difficult (6) [reviewed in Geiger et al. (7)]. The real number of infected people is most probably underestimated as it results from a mathematical extrapolation of data recorded from only partial epidemiological surveys (5, 8). In addition, wars, social conflicts and struggles, the presence of trypanosome-infected domestic animals, and climate change are recognized as factors favoring HAT development and spread (9–11). Thus, although the number estimated cases is fewer than 10,000, this disabling and fatal disease is classified among the group of poverty-promoting infectious diseases.

Two distinct forms of HAT exist which are (a) caused by two distinct trypanosome subspecies, (b) transmitted by two distinct tsetse fly vector species, and (c) widespread in two distinct geographic areas. The chronic form, caused by *T. brucei gambiense*, is transmitted by *Glossina palpalis* sp., and distributed in western and central Africa, while the acute form, caused by *T. brucei rhodesiense* is transmitted by *Glossina morsitans* sp., and restricted to East Africa. Despite these differences, the infection caused by either the chronic or the acute forms of the disease evolve similarly in two distinct clinical phases. During the first phase (stage 1 or hemato-lymphatic stage), the trypanosomes are present and multiply in the blood and in the lymph nodes; during this phase the patients exhibit intermittent fever, headache, and joint pain. Stage 2 (meningo-encephalitic stage) begins once trypanosomes have invaded the central nervous system (CNS); it is characterized by severe neurological disorders (12) [reviewed in Ref. (13)]. The two HAT forms differ in the rapidity of their respective transition from stage 1 to stage 2: several months or even several years for the chronic form, a few months or even a few weeks for the acute form. In addition, the severity of the latter is much higher than that of the former. The disease is generally fatal when not treated. Today, despite the emergence of some new drug candidates (14, 15) or drug combinations (16), the available chemotherapy remains limited and often generates severe side effects or even the development of resistant trypanosome strains (5, 17). Also, inefficient *T. b. gambiense* case detection, chronic infections that are never treated and a long stage 1 period are important contributors for stable human to human transmission in endemic areas. In contrast, for *T. b. rhodesiense* transmission, animals are the main reservoir population, greatly affecting therapeutic effectiveness and the impact of control measures (18).

*Trypanosoma cruzi* causes American trypanosomiasis, also called Chagas’ disease. This parasite is transmitted to humans and other mammals by “kissing bugs,” hematophagous insects belonging to the genus *Triatoma* [or *Rhodnius*, depending on the geographical area where the disease occurs (19)]. In addition, transfusion of infected blood, transplantation of contaminated organs, and congenital transmission are other important modes of *T. cruzi* transmission. Chagas’ disease is widespread in all South American countries affecting about 7–12 million people, and putting at risk 60–80 million others (20, 21). Three hundred thousand new cases are reported to occur each year, and 21,000 patients die annually (22). Once a host has become infected, the parasite is internalized in the cells of the innate immune system, and the infection develops progressively. Similarly to HAT, two forms of the Chagas’ disease can be distinguished. The acute form is marked by (a) the presence of *T. cruzi* trypomastigotes in the blood stream, (b) high fever, and (c) a severe hepatomegaly. By contrast, in the case of the chronic form of the disease, there are far fewer parasites present in the blood stream, and the other symptoms are also less severe. The chronic form can also be “silent” that is, in the absence of any symptom, the infection may remain undiagnosed. Nevertheless, 10–20 years later, 5–10% of these people will develop anatomical and functional abnormalities at their esophagus and their colon, while ~30% will develop myocarditis, leading to heart failure or sudden death (23).

Leishmaniasis is estimated to affect 12 million people in 98 countries, while ~350 million live in disease-risk areas (24), and presents an incidence of around 2 million cases per year. Despite more than 500,000 new VL cases per year causing the death of more than 50,000 patients (24, 25), this disease is classified among the neglected tropical diseases. In 2010, WHO estimated the disease to cause the loss of around 2.4 million disability-adjusted life years per year (24) [reviewed in Geiger et al. (7)].

*Leishmania* spp. are transmitted by sandflies belonging to the genera *Phlebotomus* and *Lutzomyia*. They induce several forms of disease in humans, ranging from localized cutaneous lesions to VL. VL, the most severe form of Leishmaniasis, is caused by parasites of the *Leishmania donovani* complex (*Leishmania donovani, Leishmania infantum*, and *Leishmania chagasi*) [see the review by Gupta et al. (26)]. Once the mammal host is infected, the parasite differentiates intracellularly inside MFs and disseminates from the skin to the spleen, liver, and bone marrow MFs (27). Most patients infected with *L. donovani* and *L. infantum* develop asymptomatic chronic latent infections. However, ~10% of infected people develop fever, severe hepatosplenomegaly, pancytopenia, cachexia, and a hyper gamma-globulinemia leading to the death if untreated (28, 29).

**Figure 1:**
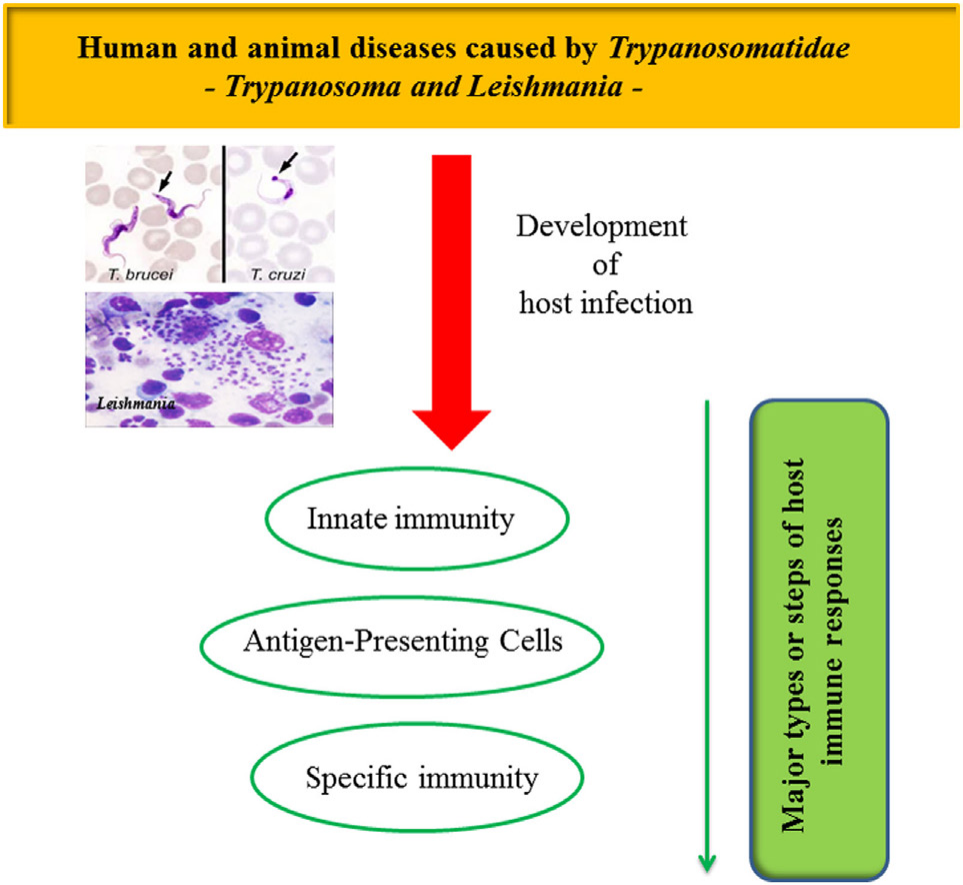
**Mechanisms used by trypanosomatids to escape their host deleterious immune response**.

In this review, the authors aimed to summarize the mechanisms trypanosomatids use to escape their host deleterious immune responses. It will focus on the aspects of the parasite–host–vector life cycle; on the host innate immunity and the key steps allowing trypanosomatids to invade their hosts; on the deregulation of antigen-presenting cells (APCs); on the disruption of specific immunity, as well as on the use of immune responses to favor parasite proliferation (Figure 1).

## Life Cycles: Parasites–Hosts–Vectors, Common and Divergent Points

The parasites’ life cycle can be divided into two crucial phases allowing the survival inside the hosts (vertebrate and invertebrate). Immediately after their transmission by the insect vector (Box 1), parasites have to resist innate immunity and develop either intracellularly (*Leishmania* and *T. cruzi*) where the parasites are no longer flagellated, or extracellularly in the blood flow (bloodstream forms of *T. brucei*). The diagnostic stage of the parasites relies on the presence of bloodstream forms of *T. brucei gambiense*, or amastigotes of *Leishmania* and *T. cruzi* in the vertebrate host (5, 30, 31). Parasite dissemination in their mammalian host occurs after lysis of the host cells (*Leishmania* and *T. cruzi*), then both intracellular amastigotes of *Leishmania* and bloodstream trypomastigotes of *T. cruzi* and *T. brucei* sp. are spread via blood circulation.

Box 1 **Transmission of parasites belonging to the Trypanosomatidae family.**

*Sandfly*: Sandflies belong to the insect order Diptera, suborder Nematocera. Within this suborder the family Psychodidae includes biting sandflies in diverse genera and non-biting owl-midges or moth flies (genus Psychoda). Among the existing phlebotomine genera, two have been proven to be vectors of one of the main zoonotic pathogens worldwide, the protozoan parasite *Leishmania*. These belong to the genera Phlebotomus in the Old World and Lutzomyia in the New World. Out of more than 800 recognized sandfly species, ~464 species are found in the New World and 375 in the Old. Among these species, only 34 are proven vectors and overall 74 species play a substantial role in *Leishmania* transmission.

*Tsetse fly*: Tsetse flies belong to the insect order Diptera, suborder Cyclorrhapha. They compose a family of their own, Glossinidae, which is placed within the Hippoboscoidea due to the morphological and reproductive similarities of tsetse flies to keds and other hippoboscid flies. Glossinidae includes the single genus Glossina with 23 species, 6 of which are further divided into 14 subspecies. Glossina species are arranged in three subgenera – Austenina, Nemorhina, and Glossina – which correspond roughly to groups of species found in different ecological settings.

*Triatomine bugs*: The members of the Triatominae belong to the insect order of the Hemiptera and the Reduvidae subfamily. Reduvidae are also known as kissing bugs, assassin bugs, or triatomines. Most of the 130 or more species of this subfamily are hematophagous and all triatomine species are potential vector of the Chagas disease parasite *Trypanosoma cruzi*. Nevertheless, only those that are well adapted to living with humans are considered important vectors (Triatoma infestans and Rhodnius prolixus).

**Figure 2:**
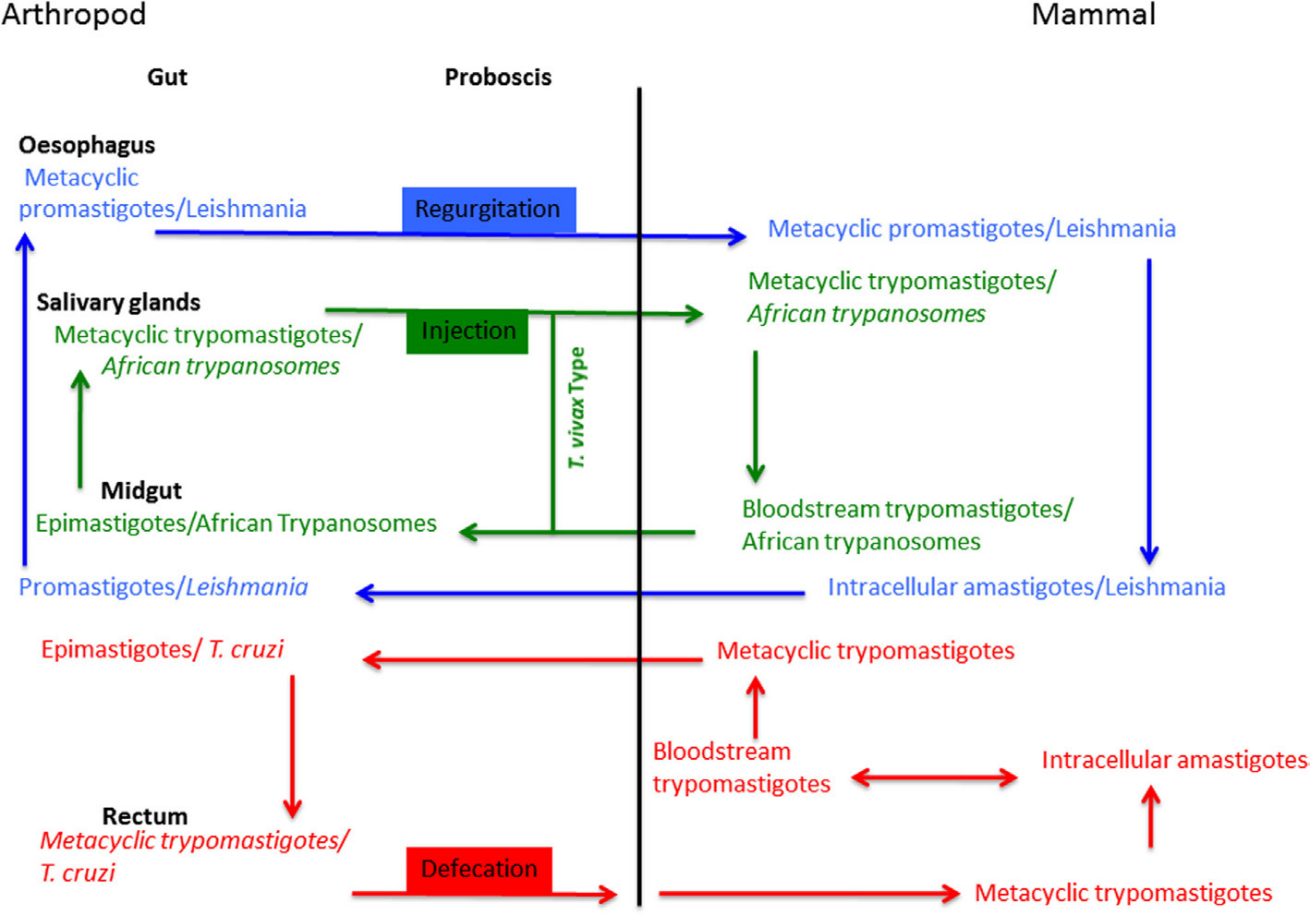
**Trypanosomatid parasites life cycles**.

Transmission from the infected host to the arthropod vector occurs when sandflies, triatomine bugs, or tsetse flies take a new blood meal, ingesting either infected cells (*Leishmania*) or free-living parasites (bloodstream trypomastigotes of *T. cruzi* and *T. brucei gambiense*). After accomplishing their intravectorial differentiation, trypanosomatid parasites have the ability to colonize various parts of the arthropod vector’s alimentary tract. Some of them are restricted to a single compartment of the alimentary tract, i.e., *Trypanosoma vivax* (Figure 2), while others have a more complex life cycle, such as *Leishmania*, which implies passage through different compartments of the alimentary tract. The transmission of Trypanosomatidae parasites by the blood feeding arthropod occurs in three ways: regurgitation (*Leishmania*/sandfly), defecation (*T. cruzi*/triatomine bugs), or injection (African Trypanosomes/Tsetse) (Figure 2). The first phase of arthropod colonization takes place in the probocis of the arthropod, or in the insect’s midgut. Once inside the midgut of the arthropod vector, parasite movements are initially restricted by the peritrophic membrane that surrounds the bloodmeal during the digestive process.

Intracellular amastigotes of *Leishmania* are released from the host cell during the cell breakage process and then differentiate into procyclic promastigotes. Depending on the subgenus, once promastigotes have been released after the destruction of the peritrophic membrane, *Leishmania* attach to the intestinal epithelium and colonize the intestine after the pylorus or adhere to the region near the pylorus. Then, the parasites migrate forward to the stomodeal valve where they differentiate into metacyclic promastigotes ready to be transmitted during a new blood meal.

For *Trypanosoma* species, the intravectorial cycle is more complex. Trypomastigote forms of *T. cruzi* change into epimastigotes inside the triatomine vector. After this process, parasites inside the midgut of the arthropod begin to multiply concomitantly with the destruction of the peritrophic membrane. Then, a second colonization phase takes place: *T. cruzi* reaches the rectum and changes into infective metacyclic trypomastigotes that can be transmitted to a mammalian host by defecation.

Bloodstream trypomastigotes of *T. brucei* or *Trypanosoma congolense*, for example, change into procyclic trypomastigotes inside the tsetse fly. Briefly, trypanosomes of the *brucei* group (*T. b. brucei*, *T. b. rhodesiense*, or *T. b. gambiense*) are carried to the gut, later passing forward to the proboscis, from where they enter the hypopharynx and reach the salivary glands, where the infective form are produced. For the *T. vivax* type, trypanosomes migrate forward to the food canal of the proboscis where they multiply. Later, infective forms reach the hypopharynx; at this stage new hosts can be infected when tsetse flies feed.

During pathogen transmission by arthropods, immediately after vessel laceration, platelets form a plug locally and produce clotting and vasoconstrictory molecules. Because vertebrate homeostasis and inflammation is complex, the saliva of hematophagous insects adapted accordingly, containing dozens of active compounds (32, 33) [reviewed in Ribeiro et al. (34)]. The nature of arthropod feeding modes is thought to have evolved independently in several insect orders and families, with the salivary composition among insects being typical of a convergent evolution scenario (35). During the transmission of Trypanosomatidae parasites by their arthropod vector, some protein and chemical components of arthropod origin are, therefore, co-transmitted to the mammalian host. They can then interfere and promote the colonization process of trypanosomatid parasites. The composition of the biological material that is co-injected (African trypanosomes and *Leishmania*) or deposited on the skin of the host (American trypanosomes) is different in its nature. If we consider the way in which *Leishmania* or African trypanosomes are transmitted, the injected cell-free biological material, along with infective parasites, contains a large amount of salivary gland proteins. In the case of *T. cruzi* transmission by triatomine, additionnal proteins, peptides, and chemicals in the feces of the bugs might also be present during the transmission of *T. cruzi*. In this particular case, the invasion of the host by infective parasites occurs later, through the bite wound or via mucosal membranes after the instinctive scratching behavior.

A brief overview of the protein salivary constitution found in the three arthropods is given in Table 1. Among the salivary components, only enzymes that belong to the Apyrase/5′Nucleotidase family, Protease family, various protease inhibitors, and the Antigen 5 family of proteins have been found to date to be present in the transcriptome and/or the proteome of all the arthropods involved in the transmission of trypanosomatids parasites. A second series of protein families has been commonly identified in sandflies and tsetse flies or sandflies and triatomine bugs; surprisingly none seems to be common to both tsetse flies and triatomine bugs. Lastly, many protein families are found specifically in the transcriptome and/or proteome of tsetse flies, sandflies, or triatomine bugs. For a vast majority, they play a role in vasodilatation, like the Maxalidan found in sandflies, Triafestin or Dipatelodipin found in triatomine bugs, or the PGE2 synthase found in the transcriptome of the tseste fly (see Table 1). In addition, proteins with anti-clotting activity are supported by different families of proteins in the three vectors of trypanosomatid parasites. Interestingly, the saliva of triatomine bugs contains a large number of proteins belonging to the lipocalin family (e.g., salivary lipocalin-5), which are described to be involved in interactions with the host’s immune response (36). Interestingly, they are also found in the transcriptome of the digestive tract, more precisely in the rectum of triatomine bugs (*Rhodnius prolixus*) (37). In addition, the lipocalin signature is also found in the extracellular material of *T. cruzi* (Sereno and Mathieu-Daudet, unpublished results). Altogether, this suggests that redundant activity supported by the protein member of the lipocalin family is required to interfere with the complex immune response that is activated during *trypanosomatids* infection.

**Table 1:** **Salivary proteins of arthropods**.

Name	Function	SF	Ts	Tr	Reference
Apyrase/5′Nucleotidase	Hydrolyze ATP into ADP, which is an inducer of platellet agregation	X			(34)
			X		(38)
				X	(39)

Proteases	Hydrolysis of peptide bonds				(34)
– Metalloprotease		X	X		(39)
– Serine protease				X	(38)

Protease inhibitor domains	Interact with the proteolytic cascade of the host homeostatic and inflammatory processes				(34)
– Serpstands for serine protease inhibitor		X			(38)
– Kazal domain				X	(40)
– Thrombinhibitor			X		(39)
– Kunitz domain			X		(39)

Antigen 5-like	Unknown	X			(41)
		X	X		(42)
		X		X	(38)

Endonuclease	Endonucleases are enzymes that cleave the phosphodiester bond with a polynucleotide chain	X	X		(34)

Hyaluronidase	Hyaluronidase hydrolyzes components of the skmatrix				(39)

Adenosine deaminase purine hydrolase	Hydrolization of adenosine into inosine and then hypoxanthine plus ribose. Adenosine and inosine induce mast cell degranulation and trigger itching reaction	X		X	(34)
		X		X	(39)

Phospholipase	Hydrolysis the platelet agregation factor	X	X		(39)

33 kDa familly	FXa clotting inhibitor	X			(34)
Nitrophorin		X		X	

15–17 kDa familly	Unknown	X		X	(34)
					(38)

Pyrophosphatase/Phosphodiesterase	Hydrolyze dinucleotides that are important inflamatory mediators	X			(34)

Glycosydase	Carbohydrate catabolism	X			(34)

Antimicrobial peptides		X			(34)

Odorant binding protein/D7 superfamilly	Antagonize inflammation and hemostasis	X			(34)

Yellow phlebotominae family	Dopachrome convertase activity	X			(34)

41.9 kDa superfamilly	Unknown	X			(34)

Maxadilan	Vasodilatator	X			(34)

27–30 kDa	Unknown	X			(34)

Possibly multigenic Glossina-specific salivary secreted protein	Unknown		X		(39)

GE-rich salivary proteins	Unknown		X		(39)

Glycine-proline rich familly	Unknown		X		(39)

Fat body and salivary 20 kDa family	Unknown		X		(39)

3–6 kDa salivary peptide	Unknown		X		(39)

Ribonucleases	Catalyze the degradation of RNA		X		(39)

Exonucleases	Endonucleases are enzymes that cleave the phosphodiester bond at the 5′ or 3′ end of the chain		X		(39)

ProstaglandE2 Synthase	PGE2 synthesis which is a vasodilatator		X		(39)

Nitric oxyde synthase	Synthesis of nitric oxyde: vasodilatator		X		(39)

Thioester containing protein	Has a reactive cysteine that can form a thioester bond to other, Pathogen, molecules		X		(39)

Fibrinogen domacontaining/ficolproteins	Familly of proteins having the Fibrinogen C motif and Ficoli motif		X		(39)

Inositol phosphatase	Hydrolysis of inositol phosphate and phosphoinositidesubstrates involved cellular process related to signal transduction, secretion, and cytoskeletal structure			X	(43)
				X	(38)

Peptidoglycan recognition protein	Pathogen recognition and initiation of innate defense mechanism			X	(39)

Salivary proteMYS2	Unknown			X	(38)

Lipocalin	The term lipocal means « cup of lipid »; they have the capacity to transport small hydrophobic molecules				
– RPAI	Inhibitor of platelet agregation			X	(43)
– Triplatin	Antiplatelet, vasodilatator			X	(35)
– Triafestin	Anti-clotting, antipain			X	(44)
– Pallidipin	Antiplatelet			X	(45)
– Triabin	Anti-clotting			X	(46)
– Procalin	Unknown			X	(47)
– Dipetalodipin	Antiplatelet, vasodilatator			X	(48)
– Nitrophorin	Antihistamine			X	(32)

## Facing Innate Immunity: Key Steps for Trypanosomatid Invasion

Many protozoa cause chronic infections, most probably owing to the millenar coevolution between parasites and host immune system. The ability to escape and/or modulate both innate and adaptive immune responses is crucial for their survival (Box 2) [in Lopes et al. (49)]. Parasites have to manipulate host cells in order to avoid the production of antimicrobial molecules and to benefit from growth factor production. Protozoa have evolved specific mechanisms to evade these defenses.

Box 2 **Innate immune responses.**

Innate immunity is based on the recognition of pathogen-associated molecular pattern molecules (PAMPs), which are present in diverse organisms, but are absent in the host and function as an exogenous signal that alerts the host to the presence of pathogens. During infection, PAMPs are recognized by pattern-recognition receptors (PPRs) that initiate signalling cascades, which lead to the activation of transcription factors in innate immune cells and have an influence on T-lymphocyte differentiation and functions.

The survival and transmission of pathogenic protozoa depends on their ability to evade or subvert host’s innate and adaptive immune responses. Evasion of innate immunity by parasitic protozoa is a critical step in their host interaction. Innate defenses include the epithelial barrier of the skin, the alternative complement cascade and other lytic serum components, lysosomal hydrolases, toxic oxygen and nitrogen metabolites of phagocytes, and immunoregulatory functions of dendritic cells (DCs). Trypanosomatids have evolved specific mechanisms to evade these defenses. The ability to avoid attack by soluble antibodies that neutralize the invasion and opsonize parasites for phagocytosis is of particular importance to extracellular parasites, such as African trypanosomes. The major strategy for evading antibody responses is the antigenic variation that protects African trypanosomes from immune recognition. The adoption of an intracellular life style, as is done by *Leishmania* and *Trypanosoma cruzi*, is the simplest way of evading humoral response. Intracellular protozoa have a remarkable adaptative capacity as they are able to resist killing by remodelling the phagosomal compartments where they reside and by interfering with the signalling pathway that leads to cellular activation. In addition, there is abundant evidence that these protozoan infections actively regulate adaptative T-cell responses, resulting in suppressed effector functions.

A great challenge to research in immunology and parasitology is the development of strategies that foster immunity against protozoan parasites and prevent their evasion, chronic or recurrent infections, and associated pathologies. A better understanding of the evasion mechanisms employed by the parasite is necessary. In the near future, a combination of strategies aimed at both early killing of parasites and neutralizing suppressive mechanisms could be necessary for effective therapies and vaccines.

### Evasion of Innate Immunity

After entering a susceptible mammalian host, protozoan parasites are targeted by pre-existing soluble factors that can potentially recognize and destroy invading parasites or target them for killing by effector cells. Serum components, such as the complement system activation, provide the first line of defense. Alternative complement activation is stimulated by non-self surfaces, such as those of pathogens, wherein the activation of C3 molecules occurs through a proteolytic cleavage promoted by C3 convertases, producing C3b molecules that bind covalently to the activator surface. These molecules subsequently promote the assembly of the membrane attack complex (MAC), which is responsible for membrane lysis (50). *Leishmania* procyclic promastigotes or *T. cruzi* epimastigotes are highly susceptible to complement action, whereas the infective metacyclic and bloodstream stages are resistant (51, 52).

*Leishmania* can evade lysis by complement by targeting host cells through complement activation. Expression of a modified surface lipophosphoglycan (LPG) (53) was found to enhance the synthesis of surface proteinase gp63 (54) and PSA-2 (55) preventing insertion or deposition of the lytic C5b-C9 complex, thereby enhancing tolerance of complement-mediated lysis (CML). Some mutants of *Leishmania major* (null-mutants for the referred molecules) were shown to have less virulence in BALB/c mice and high susceptibility to complement lysis (56, 57).

*Trypanosoma cruzi* blood forms can also survive complement activation as they express glycoproteins such as gp160, gp58/68, and T-DAF. These proteins can bind to C3b and C4b, which allow evasion of complement (58–60).

In humans, only *T. brucei gambiense* and *T. b. rhodesiense* can develop infection, as other trypanosomes are susceptible to two serum complexes with a lytic activity against trypanosomes (TLF 1 and TLF 2) (61). Despite their differences, both complexes contain apolipoprotein L1 (APOL1) (62). APO L1 in TLF1 is taken up through endocytosis via the haptoglobin–hemoglobin parasite surface receptor. *T. b. gambiense* and *T. b. rhodesiense* escape APOL1 trypanolysis by expressing distinct resistance proteins (63). The *T. b. gambiense*-specific gene, *TgsGP*, is essential for human serum resistance as deletion of *TgsGP* in *T. b. gambiense* renders the parasites susceptible to human serum and recombinant APOL1. Reintroducing *TgsGP* into knockout parasite lines restores resistance (64). Protozoa must also evade other mediators of innate immunity besides to the complement.

### Evasion of Cellular Innate Immunity

#### Remodeling Host Cell Compartments by Intracellular Parasites

*Trypanosoma cruzi* surface proteins, such as gp82 and gp35/50, first adhere to host cell surface receptors inducing calcium-mediated signaling (65, 66). Afterwards, *T. cruzi* trypomastigotes actively invade mammalian cells and their survival is dependent on their ability to subvert a calcium-regulated lysosomal exocytic pathway (67). They escape to the cytoplasm after a short period in the parasitophorous vacuole, which is necessary for the differentiation of trypomastigotes into amastigotes, triggered by the low vacuole pH (68). *T. cruzi* growth and development cannot be sustained within the parasitophorous vacuole. However, vacuole lysis and escape into the cytosol require exposure to this acidic environment, which is essential for the activity of Tc-TOX, a molecule secreted by the parasite. This molecule is active at acidic pH and forms a membrane pore, an activity which is facilitated by a trans-sialidase present on the trypomastigotes’ surface (69, 70). Another lysosome-independent route of host cell invasion has been described using the PI3K-dependent pathway (66, 71).

The initial binding and internalization of *Leishmania* promastigotes by MFs (72) is associated with/implicates the receptor-mediated classical endocytic pathway. This pathway involves a wide diversity of receptors, opsonic or pattern-recognition, such as CR3, CR1, Fc receptors, or lectin receptors such as the mannose fucose receptor [mannan-binding protein (MBP)] and the integrin family (73, 74). LPG, the main promastigote glycoconjugate, plays an essential role in promastigote adhesion to MFs, rapidly fusing with lysosomes, transiently inhibiting phagosome maturation (75) and generating a parasitophorus vacuole that maintains an acidic pH and hydrolytic activity. This delay provides enough time for promastigotes to differentiate into more hydrolase-resistant amastigotes. The replicating amastigotes ultimately survive and reside within phagolysosomes by producing glycoconjugates that are secreted or linked to surface of cell, such as GIPLS and proteophosphoglycan (PPG). These proteins protect parasites from proteolytic damage (76). A recent study shows that interaction between *Leishmania* and MFs depends on the polarization of the MF and on the CLR protein family (77).

African trypanosomes, by opposition to other protozoan parasites, never enter the cells of the host but live extracellularly in its fluids. These parasites are constantly exposed to the host’s immune monitoring so they have developed the antigenic variation mechanism, wherein they change their surface proteins to prevent elimination (78). This surface coat is made of a densely packed array of GPI-anchored variable surface glycoproteins (VSG). GPI anchors are cleaved by parasite phospholipase C (PLC) (79), resulting in the release of surface VSGs and induction of a pro-inflammatory response in cells playing a major role in innate immunity (80, 81). During early infection, the shedding of soluble VSG glycoproteins by PLC induces a polarized Th1 cell response and IFN-γ production; however, in later stages of infection, the prolonged release of these proteins inhibits MF intracellular signaling and activation (82). Antigenic variation exhibited by African trypanosomes remains their central immune escape mechanism developed during infection (83).

#### Interfering with Macrophage Functions and Host Cell Signaling Pathways

*Leishmania sp*. and *T. cruzi* are able to resist the antimicrobial mechanisms induced in phagocytic and even in non-phagocytic host cells.

During the acute phase of infection, *T. cruzi* replicates extensively and releases immunomodulatory molecules (GPI-mucins, trans-sialidase, glycoinositolphospholipids GPILS, the cysteine proteinase cruzipain), which play a major role in subverting the host’s innate immunity. GPI-mucins are responsible for parasite surface variability, leading to differential tissue adherence and evasion of host innate immune responses. Moreover, they render DCs dysfunctional for protective responses (84). *T. cruzi* uses several other mechanisms to escape immune responses from the host. In fact, the pathogens that are unable to synthesize sialic acids might adsorb these from the host as a way to engage the inhibitory siglecs, sialic acid-binding immunoglobulin-like lectins, surface proteins present in several immune cells that bind to sialic acid promoting adhesion and signaling (85). Such sialic acid–siglec association plays an important role to subvert host’s immunity [review in Khatua et al. (86)]. To escape the immune responses of the host, *T. cruzi* manipulates the CD8+ T-cell sialylation (86). When sialic acids–siglec interact, activated CD8+ T cells remain unable to kill targets that bear *T. cruzi* epitopes (87). Interestingly, recent findings propose a siglec-mediated CD33 suppression pathway of cellular function in *Leishmania* infection also (86). When, sialic acids–siglec interact, activated CD8+ T cells remain unable to kill targets which bear *T. cruzi* epitopes (87). Interestingly, recent findings propose a siglec-mediated CD33 suppression pathway of cellular function in *Leishmania* infection also (86). The evasion mechanism involving *T. cruzi* GIPLs results in the suppression of CD4+ T-lymphocyte activation (88). The cysteine proteinase cruzipain produced by *T. cruzi* is able to induce both IL-10 and TGF-β secretion and arginase expression in MFs resulting in increased replication (89). These evasion mechanisms allow the parasite to delay specific responses mediated by effector T-cells. In chronic infection, the parasite hijacks the host’s TGF-β pathway and maintains, consequently, the same rate of parasite death and replication (90). In fact, the vaccine efficacy against *T. cruzi* is called into question as this parasite is able to coexist with the immune response developed by CD8+ T cells.

Persistence of *Leishmania* and infection progression are caused by the inability of phagocytes to elicit both effective innate and adaptative responses (76). *Leishmania* alters some biological functions (disruption of cholesterol dynamics, alteration of the DNA methylation status of many host genes with antimicrobial functions, and retention of intracellular iron) to promote parasite growth (91). *Leishmania-*induced MF dysfunctions are related to the loss of microbicidal (NO, oxygen intermediates) and immunological activities (IL-1, IL-12, MHC, IRF7, and TLR2) (92, 93). These dysfunctions are correlated with the alteration of several phagocyte signaling events dependent on Ca2+, protein kinase C (PKC), mitogen-activated protein kinase (MAPK) and Janus kinase 2 (JAK2) (94). JAK2 phosphotyrosine-based signaling cascades are particularly important since tyrosine phosphorylation has been shown to play a critical role in IFNγ-inducible MF function regulation, inhibited by *Leishmania* infection [e.g., nitric oxide (NO), major histocompatibility complex (MHC) II, Interleukin-IL-12] [review in Forget (95)]. Moreover, the role of tyrosine-specific phosphatase SHP-1 in *in vivo* and *in vitro* survival of the parasite and in MF inhibition (96) was shown by the use of tyrosine kinase inhibitors (such as PTP SHP-1) that inhibit the phosphorylation mediated by the enzyme (96). However, in infected MFs, the inhibition of transcription factor STAT1α is not due to SHP-1, but probably to specific proteasomal degradation of the protein (97, 98). Another important aspect in initial establishment of infection is the presence of dead parasites in the inoculum with exposed phosphatidylserine, which facilitates uptake by phagocytes and induces TGF-beta production and TNF-alpha downregulation (99).

Like other trypanosomatids, African trypanosomes divert the MF inducible metabolism of l-arginine (100). At the beginning of infection, trypanosomes induce the arginase polyamine synthesis pathway, which decreases the production of NO, and the production of trypanothione reductase, both of which needed for host colonization and parasite growth (101). *T. brucei* releases TbKHC1, a kinesin heavy chain isoform, to stimulate the activity of arginase-1 (an IL-4Rα-independent signaling enzyme but relying on SIGN-R1-dependent IL-10 secretion) for its own growth (102). Furthermore, l-arginine depletion decreases the expression of the T-cell antigen receptor ζ chain (CD3ζ), the principal signal transduction element in this receptor, impairing T-cell functions and proliferation (103). TNF-α release by MFs exerts a trypanocidal effect (104) and is limited by the activation of trypanosome adenylate cyclase. The induction of cyclic AMP release by trypanosomes into MF and the activation of protein kinase A lead to TNF-α synthesis inhibition (105).

### Lectin Pathway

Trypanosomes use several mechanisms to escape from host immunity, such as the evasion of complement through the inhibition of the classical and lectin pathways, via binding to the C3 convertase that is essential for complement lysis in all pathways and host genetic deficiencies of the complement lectin pathway (CLP) (60). Specifically, in the CLP, the host factors mannose-binding lectin (MBL) and ficolins are able to recognize and bind to parasite surface carbohydrates that lead to activation of the complement cascade (106). *T. cruzi* is able to inactivate this lectin pathway by neutralizing the binding of MBL to carbohydrate (107). MBL induces the lysis of *T. cruzi*, and a deficiency in these host molecules only moderately compromises the defense of the host against *T. cruzi* (108). The receptor C5aR or Bradikinin B2R inhibits the translocation of calreticulin to the surface of *T. cruzi* from the endoplasmic reticulum, and inhibits activation of the host CI complement component C1, thereby promoting infection by *T. cruzi* (109, 110). In this way, calreticulin acts as a virulence factor (111).

### *Leishmania* Lectin-Complement Pathway

*Leishmania* promastigotes in the bloodstream are known to activate the complement system, reported to effectively eliminate the parasite. A greater resistance to CML is observed for infective promastigotes (metacyclic) due to the production of a surface metalloprotease GP63 and several kinases (26). These parasites have evolved to take advantage of receptor-mediated phagocytosis as a way of entering target cells and, simultaneously, of manipulating MF activation (112). Upon inoculation of the vertebrate host, C3b protein binds to the parasite, who alters it to an inactive form, preventing elimination. At the same time, the inactive C3b molecule at the surface now functions as an opsonin ensuring phagocytosis through complement receptor 3 (CR3), which will in turn inhibit IL-12 production, favoring parasite growth (26). This mechanism is independent of NFκB, MAPK, IRF, and ETS (113). Several other receptors have been described to be important for cell invasion, such as the MBP, that plays a role in *Leishmania* opsonization by triggering a antibody-independent complement activation mechanism on the MF surface (114). Complement activation by *Leishmania brazilensis* on the surface of MFs allows attachment to/invasion of the host cell (MF) by the complement receptor link between the MBL and a surface LPGs of *Leishmania* (115). In *Leishmania donovani*, the mannose-fucose receptor (MFR) and the CR3 MF receptor were shown to act independently in the attachment of parasites by human MFs (116, 117). In addition, macrophagic CR3 receptors differently inhibit *Leishmania* promastigote binding during their growth phase. Lastly, other receptors may be involved in MF infection by *Leishmania*, including MR, TLRs, and FcgRS. The infection of DCs by *Leishmania* involves several receptor/ligand interactions on the cell surface, such as antibodies FcR, a component from complement/CR and proteoglycans/heparin-binding proteins (118).

The role of polymorphonuclear neutrophil granulocytes (PMN) in *Leishmania major* survival in the host cells is not fully understood (see section Control of immune cell population life and death). These cells are able to eliminate intracellular parasite quickly, except for *Leishmania major* promastigotes, which can survive inside PMN for a few days (119). *Leishmania mexicana* amastigotes residing in the phagolysosome MF produce a large quantity of PPG, which can be secreted into the tissue after the rupture of infected cells. This PPG interacts with the complement system resulting in a decrease in the hemolytic activity of serum. It may also prevent the opsonization of *Leishmania* amastigote. PPG stimulates the C cascade by the MBP pathway. Consequently, PPG induces complement activation and, thus, contributes to the pathology of *Leishmaniasis* (120).

## Deregulation of Antigen-Presenting Cells: Preventing Adaptative Immunity

The main host cells targeted by all three Trypanosomatidae are MFs and DCs, both of which play a capital role in the response of the immunitary system as they are specialized APC (Table 2). In addition, the normal functions of T cells, B cells, and T-helper cells (Th1 and Th2) involved in host immune responses, may also be modulated, more or less specifically, by the parasites.

**Table 2:** **Effects of either *Trypanosoma brucei sp*., *Trypanosoma cruzi* or *Leishmania* sp on host immune responsive cells [for *T. brucei*: Dagenais et al. (121); Vincendeau and Bouteille (122); for *T. cruzi*: Chaussabel et al. (84); Flávia Nardy et al. (123); for *Leishmania*: Bogdan et al. (124)]**.

	*T. brucei*	*T. cruzi*	*Leishmania*
**Macrophages**			

– Faulty Ag processing and inability to present Ag to T cells	+		+
– Faulty epitope association with MHC-II	+		+
– Decrease in T-cell responses	+		+
– Th2 response → NOS inhibition and activation of arginase production	+		+
– Production of NO, PG, IFN-γ, and TGF-β	+		+
TGF-β inhibits IL-4, IL-5, and IL-6 → inhibition of B cells differentiation and proliferation			
– Inhibition of caspase-3 production by DC → inhibition of DC apoptosis			+

**Dendritic cells**			

– Inhibition of MHC-II, CD40, CD80, CD86 expression and inhibition of TFN-α, IL-6, IL-10 production:	+	+	+
Inhibition of DC maturation			
No differentiation of naive T-CD4+ lymphocytes into			
Th1 (producing: IFN-γ, IL-2, TFN-α)			
Th2 (producing: IL-4, IL-5, IL-10)			
– DCs depletion	+		+
– Inhibition of caspase-3 production → inhibition of DC apoptosis			+
– Production of IL-4 → activation of Th2 response (Th2 secretes IL-4, IL-13) → activation of alternative pathway of macrophage → *Leishmania* survival	+		+

**TH1/TH2**			

– Th2 cellular response activation; production of IL-4, IL-10, IL-13, and TGF-β → inhibition of Th1 responses	+	+	+
– Th2 responses	+		+
Inhibition of macrophages NOS production			
Activation of arginase (l-arginine → l-ornithine biosynthesis)			
Biosynthesis of polyamines and trypanothione			
Favors parasite development, macrophage infection, and parasite survival			

### *Trypanosoma brucei* sp./*T. congolense*, and the Human/Animal African Trypanosomiasis

These *Trypanosoma* species, causing either human or animal African Trypanosomiases, induce a global MF and T-cell-mediated immunosuppression, as well as the development of suppressive cell phenotypes, in infected cattle or mice (125–128). In the case of MFs, both classically and alternatively activated cells may develop such suppressive phenotypes (129). They are antagonistically regulated and their development is modulated by the cytokine environment. So, while classically activated MF are induced by type I cytokines (TNF-α, IL-12, IFN-γ) and inhibited by type II cytokines (IL-4, IL-10, IL-13, TGF-β), the reverse is true for alternatively activated MF (130).

The MF and DC immune response function includes: (a) processing of parasite antigens in the endocytic pathway and (b) co-stimulation and presentation to T-helper cells (Th cells) of trypanosome immunogenic peptides (121). In early infection by *T. b. rhodesiense* (clone LouTat 1), naive VSG-specific Th cells were more activated by DCs than MF, which resulted in Th1-mediated protective responses (121). Then, the specific Th cells secrete molecules that activate both immune systems, innate and adaptive, with the aim to destroy the infecting trypanosomes. Thus, MF and DCs modulate the adaptive anti-trypanosomal immunity by controlling antigen presentation. According to Dagenais et al. (121), trypanosomes may have evolved so as to alter antigen presentation for their own survival as a consequence of the pressure exerted by the immune system. Depletion of DCs and splenic MFs co-stimulatory molecules downregulation by *T. b. rhodesiense* contributed to inability of both DCs and MFs to generate an efficient T-cell response specific to VSG. The ability to modulate MF and DC antigen presentation functions allows the parasite to escape killing by immune cells and may contribute to the overall immunosuppression occurring during trypanosomiasis (131).

Murine *T. brucei* infection was reported to affect the co-expression of processed antigens and MHC class II molecules on the plasma membranes of MFs with the consequence of a reduced ability of these cells to present antigen in *T. brucei*-infected animals (132). The permanent contact of the trypanosomes with the host immune system may have induced in some *T. brucei* strains the ability to modulate MF antigen presentation process. This process involves peptide loading onto MHC class II molecules and/or [MHC class II-peptide (pMHC)] complex translocation to the cell surface for presentation to antigen-specific Th cells (132). It was also found that suppressive MFs inhibit the proliferation of lymphocytes responding to mitogens and antigens and, thus, reduces the proliferative cytokine IL-2 secretion by T-cells (133). Moreover, the levels of IL-2 receptors on the surface of these T cells were lowered (134). However, it was also reported similar levels of immunosuppression in infected animals that are both susceptible and resistant; thus, the real efficiency of this mechanism to ensure parasite survival in its host remains questionable (135, 136).

The secretome (total excreted-secreted proteins) of *T. b. gambiense* was shown to impair the lipopolysaccharide (LPS)-induced maturation of murine DCs (131). When DCs are stimulated by LPS, MHC class II, CD40, CD80, and CD86 molecules are upregulated, and cytokines, such as tumor necrosis factor alpha, interleukin-10 (IL-10), and IL-6 are released at high levels. In *T. b. gambiense* secretome-stimulated DCs, upregulation and secretion of the previous molecules is significantly reduced. Moreover, the inhibition of DC maturation resulted in the loss of their allostimulatory capacity, leading to a dramatic decrease in Th1/Th2 cytokine production by co-cultured lymphocytes. These results provide new insights into a novel efficient immunosuppressive mechanism directly involving the alteration of DC function, which might be used by *T. b. gambiense* to interfere with the host immune responses in HAT and promote the infection process [review in Garzón et al. (131)].

### *Trypanosoma cruzi* and Chagas’ Disease

A number of reports support the idea that, during the infection process, *Trypanosoma cruzi* is able to elicit severe autoimmune responses in the host, which contributes significantly to the development of the pathogenic process of the Chagas’ disease. *T. cruzi* amastigotes escape host humoral immune responses by developing inside host cells. The parasite surface is covered with mucin glycoproteins that prevent *T. cruzi* from being recognized by the host immune system, thus favoring its establishment in its host and disease onset. These mucins are receptors of residues of sialic acid, which are detached by trans-sialidase from glycoconjugates of the host; this chemical modification is of interest as the modified mucins are even more efficient than the non-sialised glycoproteins were.

In addition, *T. cruzi* produces several complement regulatory molecules, which allow the parasite to prevent complement activation, thus allowing it to evade CML (58, 66, 137).

Moreover, *T. cruzi* induces the production of both Th1 and Th2 cytokines in infected individuals, and high expression levels have been reported for Th1 cytokines IFN-γ and IL-2, as well as for Th2 cytokines IL-4 and lL-10 (138). In *T. cruzi*-infected individuals, IL-10 gene expression is actively upregulated as indicated by the presence of significantly increased levels of the corresponding mRNA. This observation suggests that high levels of IL-10 may contribute to parasite persistence, as IL-10 is known to inhibit host protective Th1 immune responses (139). Thus, the induction of IL-10 biosynthesis may be crucial for the parasite’s survival in its host. By contrast, elimination of the parasite is largely under the control of Th1-specific cytokine production (IL-12, IFN-γ, TNF-α) (140). IFN-γ produced by NK cells during *T. cruzi* infection will activate phagocytic cells, which in turn will produce toxic reactive nitrogen intermediates that, ultimately, will kill internalized parasites (141). Despite the fact that the induction of cytokines leads to both cell-mediated and humoral response and, thus, suggested to be important for the development of effective immune responses, the susceptibility or resistance of mice to *T. cruzi* infections seems not to be related to a given cytokine response (142). Lastly, the ability of *T. cruzi* to infect a host, to survive and develop, and to cause Chagas’ disease depends on a complex balance between Th1 and Th2 cytokine production, as they display antagonistic effects, the former being protective for the host, the latter for the parasite.

### *Leishmania* sp and Leishmaniasis

Basically, the evasion strategies of *Leishmania* involve diverse mechanisms, including the capacity to survive within MFs, especially by inhibiting the oxidative burst occurring in activated MFs, and to modulate the T-cell immune response. Fixation of the complement C3 protein and the subsequent binding to CR1/CR3 is essential for the initial intracellular survival of infective-stage promastigotes (143). Once *Leishmania* are located intracellularly, after receptor-mediated endocytosis, they downregulate the active oxygen-dependent killing mechanisms of activated MFs (144) [review in Bogdan et al. (145)]. The total cellular and membrane acid phosphatase activity correlates with parasite virulence (146). LPG is another potent inhibitor of oxidative burst. It works by inhibiting PKC (147), which is the enzyme involved in the production of oxidative metabolites. *Leishmania* produces substances with the ability to scavenge the effect of oxidative metabolites. Furthermore, *Leishmania* amastigotes display high activity for enzymes that are known to degrade these toxic MF products (148). In addition, antiparasitic processes dependent on oxygen, phago-lysosomal processes, physical (low pH, osmotic stress), or biological factors (lytic enzymes) contribute to the MF antiparasitic activity. Hence, the importance of LPG, which is directed against the antiparasite effectors produced by the host MF (26, 99, 149, 150).

The activation of type 1 T-helper lymphocytes (Thl) by APCs requires surface expression of MHC class II, interaction with costimulatory receptor–ligand pairs (B7/CD28, CD40/CD40L, MHC class II/CD4), and peptide presentation by MHC class II [reviewed in Kaye (151)]. *In vitro* studies demonstrated the implementation of various mechanisms *Leishmania* uses to impede T-helper cell responses. It was first demonstrated that *L. donovani* amastigotes interfere, at the transcriptome level, with the MHC class II upregulation by IFN-γ [reviewed in Reiner (152)]. In addition, *Leishmania* was shown to be able to downregulate MHC class II expression (153). In contrast to other intracellular microorganisms (such as *Listeria monocytogenes*), *L. donovani* does not upregulate the production of B7-1 costimulatory molecules and the heat-stable antigen (HSA) in MFs. The MFs were not susceptible to stimuli, that normally upregulate B7-1 or the HSA, as does *Listeria* infection or the administration of LPS, of IFN-y or of a crude mixture of mitogen-activated T-cells cytokines (154). Another critical aspect for Th-cell activation, in addition to the presence of MHC class II and costimulatory molecules, is the availability of parasite-derived peptides for loading onto the MHC molecules (155) [review in Bogdan et al. (145)].

Although *Leishmania* parasites interact with multiple cell types, MFs and DCs are clearly the most important cells influencing the infection progression and outcome. Interleukin 12 (IL-12) is a critical cytokine necessary for CD4+ Th1 development and IFN-γ production (156) [review in Dong and Uzonna (157)]. Although MFs are able to phagocytize *Leishmania* efficiently, their ability to produce IL-12 is selectively impaired by the parasites (158). In addition, infection of MFs by *Leishmania* also leads to the production of immunoregulatory cytokines, such as IL-10 and TGF-β, which are known for their ability to inhibit or deactivate MF functions (see Evasion of Innate Immunity) (159).

Several reports show that DCs, highly efficient APCs, play a central role in orchestrating immune responses in leishmanial disease (160). Although MFs are also specialized APCs, the main host cell for *Leishmania*, as well as the most efficient parasite-killing effector, infected MFs do not secrete IL-12 (161); hence, they are unable to stimulate an antigen-specific CD4+ Th1 cell response (162) [review in Dong and Uzonna (157)]. In *L. major* infection, Ritter et al. (163) demonstrated that CD8α and langerin-negative DCs are the principal APCs. They express dermal markers of DC (MHC II^high^, CD11c^+^, CD11b^+^, CD8α^−^, and CD205^low^) (163) and overcome the induction of CD4+ T-cell response. In addition, Kautz-Neu et al. (164) reported that, in the case of low dose infection, DCs may play a role in the cutaneous leishmaniasis/*L. major* pathogenesis via the induction and expansion of regulatory T cells (164–166). In this context, the production of IL-12 by APCs is of critical importance as it is able to polarize naive T cells into Th1 subset, and, subsequently, to induce IFN-γ production (161).

Lastly, intracellular signaling activation cascades that lead to the production of effector molecules are important for an effective control of pathogens in infected host cells. A number of pathogens are able to modulate signal transduction pathways to favor their survival (167). Since *Leishmania* are obligate intracellular parasites, their survival inside mammalian host cells is critically dependent on their ability to successfully disrupt host cell signaling events [review in Dong and Uzonna (157)], which would otherwise lead to the generation of killing effector molecules. To avoid killing, the parasites must be actively involved in almost every aspect/or all aspects of host cell signaling, manipulating/inhibiting from the production of microbicide molecules to the elicitation of protective cytokines.

## Disruption of Efficient Specific Immunity

**Figure 3:**
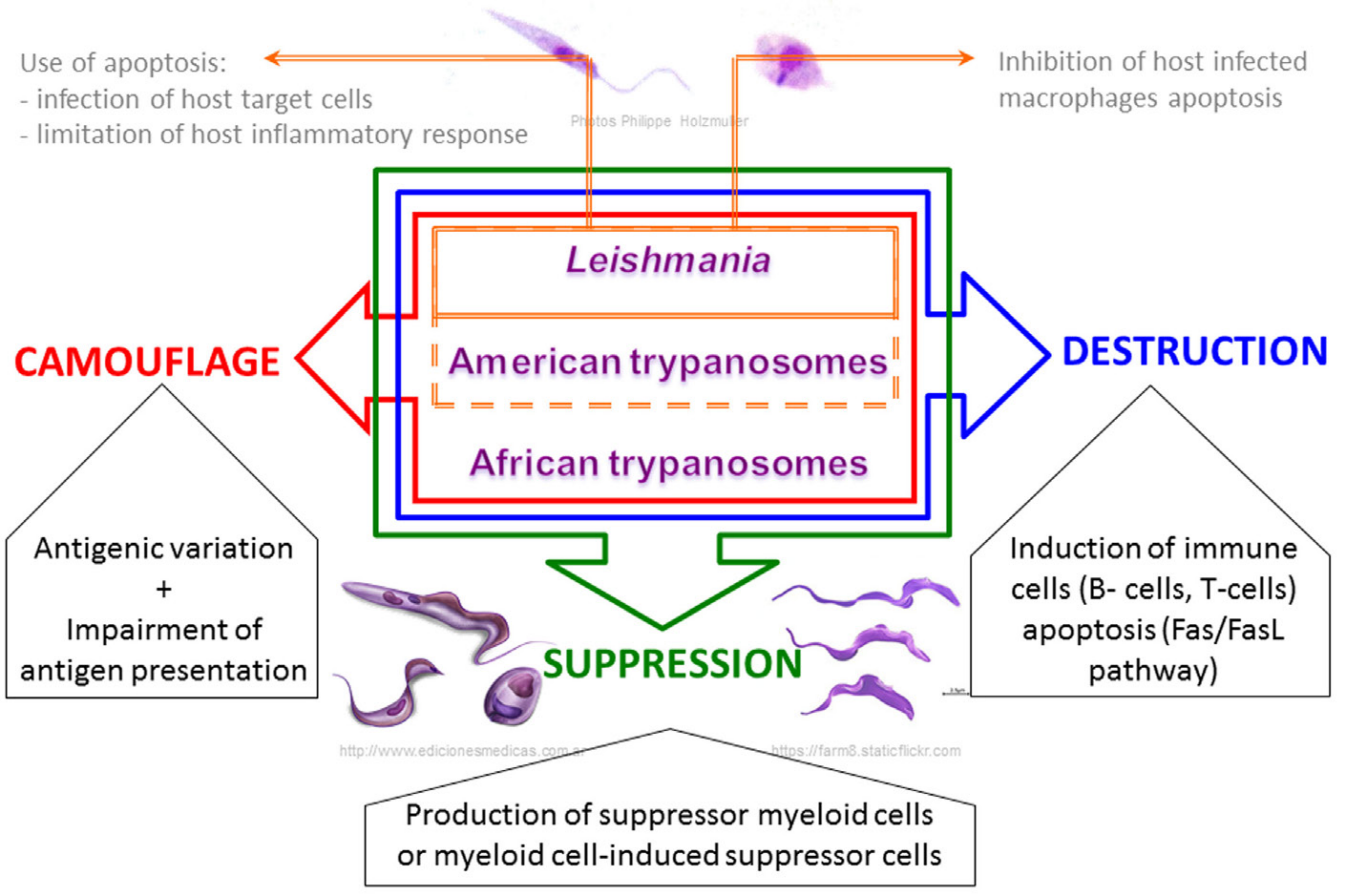
**Schematic view of the common strategic arsenal developed by trypanosomatids to interrupt effective host immunity, from immune evasion to immunosuppression**.

Although mammalian hosts have developed several immunological mechanisms to eliminate both intracellular and extracellular parasites, trypanosomatids parasites have in turn developed strategies to escape the host immune system, which enable their survival and replication. The most striking observation during infections by trypanosomatids is that specific immune responses do exist but they are completely inappropriate and ineffective, or are even responsible for immunopathological processes (168). Trypanosomatids exhibit different cellular differentiation stages and different strategies to interact with their host, but in addition to the specificity associated within the genera *Leishmania* or *Trypanosoma*, these parasites have developed common features in order to subvert their hosts’ immune system and ensure successful transmission (99, 123, 169) (Figure 3).

### Control of Immune Cell Population Life and Death

Apoptosis, a major representation of programed cell death, is an essential physiological process in maintaining cellular homeostasis, particularly in the immune system, where it participates in both eliminating autoreactive or failed cells and controlling a proliferative response (170). Trypanosomatids have developed refined mechanisms for inducing or preventing the apoptotic cell death of their hosts’ immune cells. For instance, *Leishmania* and *T. cruzi*, additionally to their use of apoptosis mimicry to invade MFs (171, 172), are able to regulate apoptosis of target cells positively or negatively. This is well illustrated for *Leishmania* with neutrophils, whose lifespan can be either extended by the parasite to benefit potentially from the protection of a safe intracellular niche (173) or reduced after parasite intake, both to limit antiparasite response (174) and fuel parasite growth (175). Neutrophils are the first phagocytes recruited to the inoculation site (176) and take part in the “Trojan horse” MF invasion, where infected apoptotic neutrophils shuttle parasites silently (i.e., without inflammatory signal) via phagocytosis to their primary host cell; *Leishmania* is able to delay neutrophil apoptosis, allowing the release of chemotactic molecules and consequent recruitment of DCs and MFs, as well as the production of TGF-beta that will contribute for the silent entry into MFs (99, 177, 178). The opposite neutrophil modulations are linked to different parameters, such as the genetic background of the animal model used or the parasite inoculation route (179), but also the molecular pathways modified, such as inhibition of pro-caspase-3 processing (173), or activation of the survival pathway involving extracellular kinases (ERK1/2) (180).

Although they all subvert MF activity during infection, targeting the core cell type of the mammalian host immune system, trypanosomatids behave differently in managing MF cell death depending on the genus considered. *Leishmania* parasites clearly prevent MF apoptosis either when directly housed by this cell type (181), or when using the above-mentioned “Trojan horse” strategy (177, 178). *T. cruzi* also uses phagocytosis of apoptotic cells by infected MFs to promote parasite growth, upon synthesis of transforming growth factor β (TGF-β), prostaglandins, and polyamines similarly to *Leishmania* (87). As they are exclusively extracellular, African trypanosomes are constantly exposed to the hostile host environment and have, in particular, developed mechanisms to cope with the microbicidal action of MFs, without specially inducing or repressing apoptosis, but through fast induction of the alternative arginine pathway leading to polyamines production (80, 182).

One very common feature developed by trypanosomatids to ensure immune evasion consists in destroying the lymphoid lineage by parasite-driven cell death. Apoptosis of T lymphocytes during the contraction phase of an immune response occurs through re-stimulation of activated T cells in a process termed activation-induced cell death (AICD), or results from the lack of survival factors, commonly referred to as activated T-cell autonomous death (ACAD) or death by neglect (183). Using the murine infection model for *T. cruzi*, splenic CD4+ and CD8+ T-cells were shown to express CD95 (Fas/Fas ligand apoptotic pathway) 2–3 weeks post infection. This observation is in accordance with their death by AICD (184). *Leishmania* parasites use the same strategy to eliminate both CD4+ and CD8+ T-cells, as observed in active human cutaneous Leishmaniasis (185), with more apoptotic spleen and peripheral blood T-lymphocytes in infected dogs compared to control animals (186). However, the molecular mechanisms are not as well defined: expression of Fas and FasL is increased in splenic human lymphocytes in acute disease (187), and Bim, a member of the Bcl-2 family, could also be involved in the apoptosis of T-cell in mouse models infected with different *Leishmania* species (188), which can otherwise be related to downregulation of kinase activities by Ser/Thr phosphatase (189). Extracellular African trypanosomes have also developed paracrine mediators able to induce not only CD45-dependent T-cell death (190) but also memory B-cells apoptosis (191, 192), which dramatically impairs the ability of infected hosts to develop adaptative immunity. In the same way, *T. cruzi* modulates the death of both IgG(+) B cells reactive to the parasite through B-cell–B-cell killing (193) and also induces a marked loss of immature B cells in the bone marrow through myeloid cells secreting a cyclooxygenase pathway product (194), thus limiting host defense and disabling B-cell generation to favor its chronic establishment.

The above paragraphs show one of the keypoints/key aspects/key mechanisms, leading to a successful infection by trypanosomatids: the ability to subtly modulate the life and death of immune cells when interacting with the host immune system.

### Abolition of Efficient Specific Immunity

#### Molecular Camouflage and Altruistic Behavior

The most well-known system for evading the host’s specific immune response is probably the antigenic variation developed by African trypanosomes. They have a “repertoire” of variable antigenic types (VATs), trypanosome variants in a given population, and they can change this surface coating by controlling variant-specific surface glycoproteins (VSGs) gene expression. When infecting the hosts, the immune system targets the major VATs; thus, the parasites with non-targeted or new VATs evade destruction. This antigenic variation developed by the trypanosomes (several species, subspecies, types, and strains) explains why they can escape from an effective immune response developed by livestock and human populations in different geographical areas (195). Indeed, 10 million identical VSGs cover the surface of the trypanosome at any given time. On the one hand, they are highly antigenic to focus the host humoral immune response; on the other hand, they make it possible to circumvent the immune destruction of the parasite by sacrificing the majority population while maintaining an untargeted population (78). Actually, specific immunity against the trypanosome’s VSGs is effective, but delayed in time, which unfortunately allows the parasite to produce its other immunomodulatory effect on the host response. Although antigenic variation is a hallmark in African trypanosomes, other Trypanosomatidae also use this molecular mechanism to evade the host response. In *Leishmania*, for instance, the central repetitive domains of the hydrophilic acylated surface proteins (HASPs) are highly variant in their amino acid sequences, both within and between species, and clearly play a role in immune recognition in the host, albeit not yet fully resolved (196). In the same way, antigenic variation in *T. cruzi* led to the question as to whether *Trypanosoma cruzi* should be called the “cruzi” complex, as the parasite’s diversity is substantial not only among strains but also because the interaction of the different infecting clones in the host will determine the severity of the infection (197).

#### Disability of Antigen Presentation (See Also Section III APCs)

Trypanosomatids, through their complex life cycles and different parasitic stages, have also developed sophisticated strategies for interfere with antigen presentation, by decreasing the expression of MHC molecules, by inhibiting the costimulatory molecules CD80 or CD86, or the synthesis of IL-1. Accordingly, specific T-cells are less stimulated and become anergic, leading to a non-efficient or an inadequate immune response. During progressive illness caused by *Leishmania*, two concomitant phenomena have been observed: an inability of APCs to process antigens properly, and the generation of a non-functional T-cell response to the processed antigens, despite functional signaling of human leukocyte antigen HLA/MHC class II molecules to T-cell receptors (TCR) (154, 198, 199). Moreover, the inability of APCs to process antigens properly has been linked to the inhibition phagolysosome biogenesis after *Leishmania* phagocytosis. In fact, the *Leishmania* surface metalloprotease GP63 cleaves a subset of soluble receptors, *N*-ethylmaleimide-sensitive-factor attachment protein receptors (SNAREs), consequently inhibiting the MHC class I presentation of *Leishmania* exogenous antigens, resulting in reduced T-cell activation (92). The strategy developed by *T. cruzi* in restricting antigen presentation is a little different, with hyperpolarization of the presented antigen repertoire (immunodominance), avoiding complete pathogen elimination by host effector cells, and thus favoring host parasitism (200). A hallmark of African trypanosome infection is that APCs functions are substantially altered, but the weight of antigen presentation in the balance between immunosusceptibility and immunotolerance appears to be more complex than for other trypanosomatids (121). Early studies in mouse models supported reduced presentation of non-parasitic exogenous antigens to T cells, presumably due to the altered display of antigenic peptide–MHC class II complexes (127, 132, 201). However, it remains unclear how the ability to present antigen is modulated among the APCs subsets and to what extent it could affect the infection outcome. This was illustrated in resistant mice infected with *T. congolense*, which were able to control infection in an MHC class II-restricted immune response manner, but only when the IL-10 function was not impaired (202), suggesting precarious effectiveness of antigen presentation in response to African trypanosomes.

#### Trypanosomatid-Induced Imbalance of T-Cell Populations

In addition to the apoptotic cell death occurring in immune cell populations, the loss in number and functionality of T- and B-lymphocytes during trypanosomatid-induced diseases is a paradigm referred to as “exhaustion” (203, 204). In *T. cruzi* infections, the repertoire of CD8 (+) T-cells is dramatically restricted, which is a particular phenomenon known as immunodominance. The latter, despite targeting a different lymphocyte population, can be related to the response to a VAT-specific VSG during African trypanosome infections (78). Interestingly, mice that developed immune responses against subdominant/cryptic CD8 T-cell epitopes corresponding to the immunodominant antigen were significantly protected against *T. cruzi* infection (205). In the same way, exhaustion of cross-reactive responses to subdominant invariant epitopes by antigenic variation of the dominant antigens from African trypanosomes could explain the inefficiency of the selected lymphocyte populations, but at the same time question on the possibility of restoring protective cross-reactive immunity (206). In fact, in terms of lymphocyte populations, an increase in the CD4+:CD8+ T-cell ratio and IgG1 could be associated with self-cure in African trypanosome-infected natural host, whereas a decrease in the CD4+:CD8+ T-cell ratio and IgM could lead to disease development (207). Regarding *Leishmania* infections, susceptibility or resistance were associated long ago with a dichotomy in the development of immune response dominated by T-helper 2 (Th2) versus Th1, respectively. This was based on experimental data from mouse models infected with *L. major*, but it does not seem to be generalized to all *Leishmania* species, as complex early events shape the immune response (208), and especially as polarization is not observable in the natural host (human) where Th cells and CD4+:CD8+ ratio are either associated with the healing process or the development of the different clinical forms (209). Additionally, using mutant mouse models, it has been possible to explain more clearly the non-cure arising in resistant mice, which was due to a Treg cell imbalance (T-regulator cells), whose primary function is to suppress ongoing Th1 responses as to control tissue damage, and that functions as a suppressive pathway contributing to parasite persistence (210).

## Use of Immune Responses for Parasite Proliferation

To escape from host immunity, trypanosomatids interfere with the physiological function of various molecules of their host, such as arginine and calcium.

### Arginine and Trypanosomatidae

**Figure 4:**
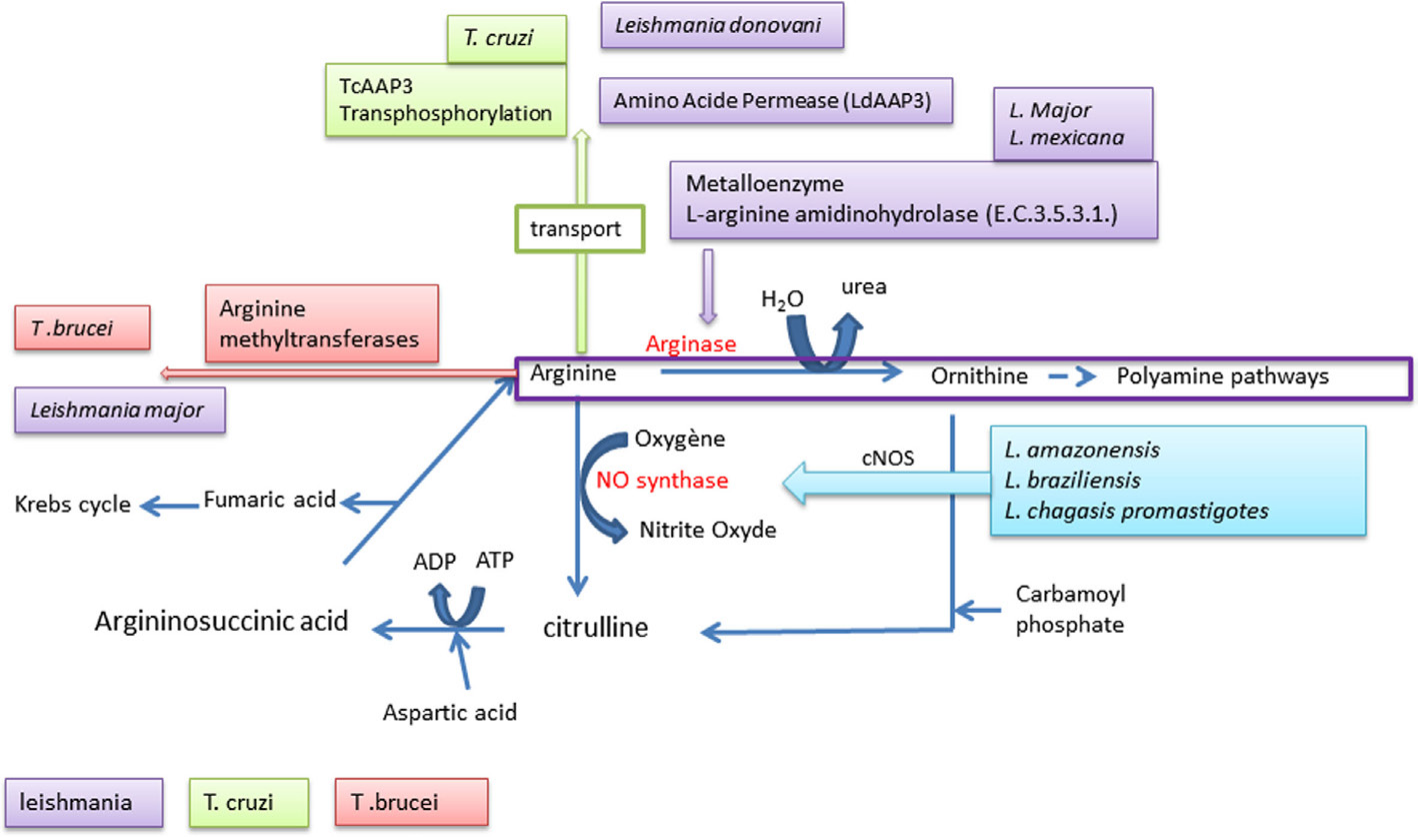
**Arginine used by Trypanosomatidae**.

To survive and multiply in their host, Trypanosomatidae have the possibility of exploiting the host metabolic machinery (Figure 4). The mechanisms used are diversified. Several strategies have been developed by Trypanosomatidae to escape host immunity. They have the potential to act upon the defense mechanism of the host, either to create a bypass of the host’s defense mechanism, such as the arginine and lectin pathway, which is a mechanism of complement evasion, or to scavenge elements produced by the host, such as calcium.

Arginine is an amino acid with a basic fundamental role in the animal kingdom as a precursor of protein synthesis. It is also the substrate for enzymes leading to the production of NO or of polyamines (211). Polyamines are known growth factor promoters, and NO is a highly potent antimicrobial molecule. Physiologically, arginine is metabolized via two pathways, including a catabolic pathway involving its degradation by arginase to generate urea and ornithine, a precursor of the polyamine pathway. These products of metabolic degradation are known to promote *Trypanosomatidae* growth; on the other hand, NO exerts a broad spectrum of antimicrobial activity and it is highly toxic for *Trypanosomatidae*. The parasites *Trypanosoma brucei* (212) or *Leishmania major* (213) interfere with these processes at various steps.

They are able to promote the arginine methylation via methyltransferases. In *Trypanosoma cruzi* (214), a transphosphorylase (TcAAP3) acts on the phosphorylation level of the host arginine, while *L. donovani* scavenges the arginine synthesized by the host through an amino acid permease (LdAAP3) (215). In addition to these processes another system is exploited by Trypanosomatidae, which consists in using the host arginine for itself. *Leishmania* can hydrolyze the l-arginine of the host by an l-arginine aminohydrolase (E.C.3.5.3.1.) allowing the parasite to escape the production of host microbicidal NO (216). A few species, such as *Leishmania amazonensis*, *Leishmania braziliensis*, and *Leishmania chagasis* promastigotes, have a significant effect on this NO production using an irreversible inhibitor of nitric oxide synthase (iNOS) (217).

### Free Calcium in Trypanosoma

**Figure 5:**
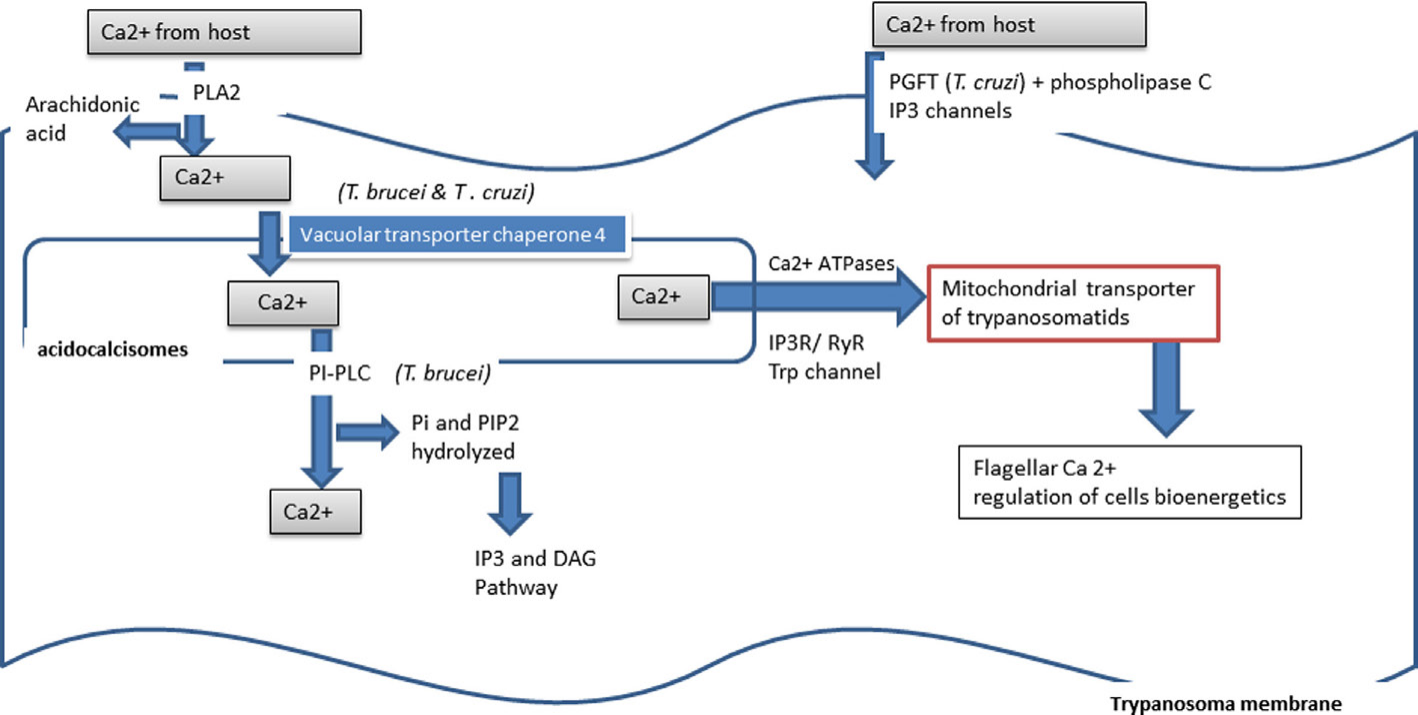
**Calcium used by *Trypanosoma***.

The free calcium ion is important for trypanosomatid survival and multiplication (Figure 5). These pathogens are able to scavenge the ion either from the host cell intracellular stock or from the host extracellular stock. In *Trypanosoma*, intracellular calcium is stocked in a peculiar cell structure called the acidocalcisome (218). Cleavage activation of the *Trypanosoma cruzi* trypomastigote factor (PGFT) activates the PLC pathway that induces the release of intracellular free calcium via inositol, 1,4,5-triphosphate (IP3) sensitive intracellular channels. Calcium release induced the reorganization of host cell microfilaments, which play a crucial role in mammalian host cell invasion by *Trypanosoma cruzi* (219). Calcium is taken up from the surrounding environment of the parasite through the activity of a Ca^2+^-ATPase (220). In *Trypanosoma*, the intracellular stock of free calcium, pyrophosphates, and polyphosphates is stored in specialized organites called the acidocalsisome (221). Ca^2+^ entry is regulated by PLA2 and activated by arachidonic acid and Ca^2+^ itself (222). Arachidonic acid appears to play a major role in calcium release from the cellular acidocalcisome (223). Arachidonic acid and the melittin peptide, of amphiphilic nature, induce an increase in intracellular calcium concentration in procyclic *Trypanosoma brucei*, *Leishmania donovani* promastigotes, and *Trypanosoma cruzi* amastigotes. In *Trypanosoma cruzi*, calcium plays a role in flagella motility via the flagellar calcium-binding protein (FCaBP), in all stages of development. This calcium-binding protein is localized in the flagellar membrane and acts in a calcium dose-dependent manner for its activity. In *Trypanosoma brucei* and *Trypanosoma cruzi*, calcium is carried by vacuolar transporter chaperone 4 (224). In *Trypanosoma brucei*, calcium release from intracellular storage acts through the activation of phosphoinositide phospholipase C (PI-PLC), which hydrolyzes phosphatidylinositol (PI) and PI 4,5-biphosphate (PIP2), involving the inositol 1,4,5-triphosphate (IP3)/diacylglycerol (DAG) pathway for this activation (225).

### Calcium Ion in *Leishmania*

**Figure 6:**
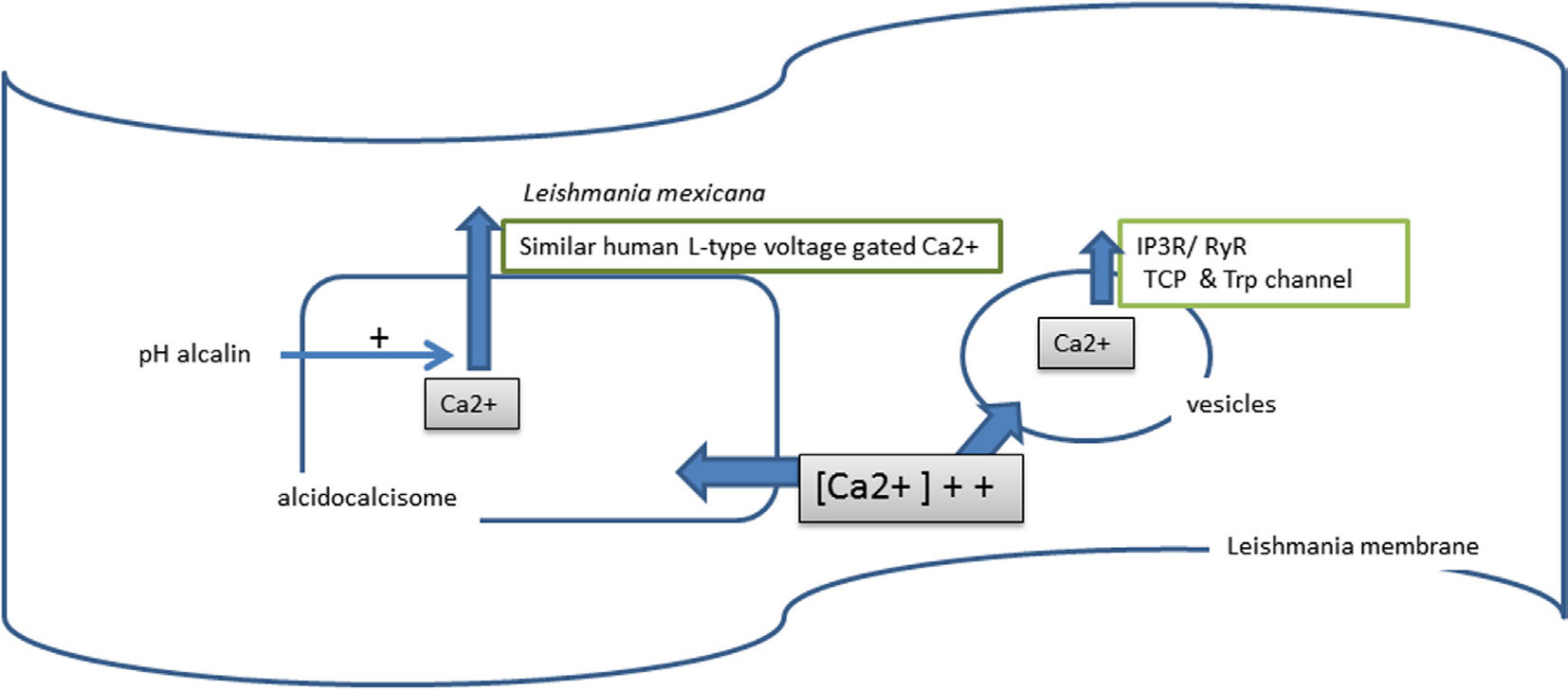
**Calcium used by *Leishmania***.

In *Leishmania*, calcium is stored in two cellular compartments: vesicles in mitochondria (226) and in the acidocalcisome (218) (Figure 6). The osmotic regulation of intracellular calcium is crucial for parasite survival and involves a set of ATPases whose location varies from the plasma membrane to the sarco/endoplasmic reticulum (227).

During infection of the MF, a burst occurs in the calcium steady-state concentration (228). During this process, free calcium from vesicle storage is released by the action of IP3 receptors, ryanodine receptor channels (RyRs), two-pore Ca(2+) channels (TPCs), and intracellular transient receptor potential (Trp) channels, which are mammalian channel homologs (229). This disturbance in intracellular calcium concentration has several consequences: (i) activation of guanylyl cyclase, which increases parasite infectivity (230); (ii) the release of calcium from the acidocalcisome, due to alkalization of the cytoplasmic pH (231); (iii) depolarization of the mitochondrial membrane potential and induced ATP loss, generating *Leishmania* cell death by apoptosis (232). Calcium channel blockers and nucleotides (UTP) possess anti-*Leishmania* activity (233, 234).

## Conclusion

In conclusion, *Trypanosomatidae* parasites are multistage organisms that require a vertebrate host and an insect vector, in which they undergo many cell differentiations. Designing drugs that persistently interrupt the life cycles of these parasites requires a comprehensive understanding of their biology and the mechanisms involving host–vector–parasite interactions. Owing to the difficulties in controlling diseases caused by trypanosomatids, many studies have been focused on the strategies developed by the different parasites to escape host immune defenses, with a view to characterizing weaknesses in their escape processes that could be used to fight them. The goal of our review is to focus on these strategies developed by the different parasites to escape host immune defenses to improve knowledge of these interactions in order to initiate novel strategies for controlling the diseases caused by *Trypanosomatidae* parasites.

## Author Contributions

AG, GB, DS, JP, J-LL, PV, and PH wrote the review manuscript, read, and approved the final manuscript.

## Conflict of Interest Statement

The authors declare that the research was conducted in the absence of any commercial or financial relationships that could be construed as a potential conflict of interest.

## References

[1] Stuart K Brun R Croft S Fairlamb A Gurtler RE McKerrow J Kinetoplastids: related protozoan pathogens, different diseases. J Clin Invest (2008) 118:1301–10. 10.1172/JCI33945 18382742 PMC2276762

[2] WHO. WHO Technical Report Series Control of the Leishmaniases. (2010). Avaialble from: http://apps.who.int/iris/bitstream/10665/44412/1/WHO_TRS_949_eng.pdf

[3] Welburn SC Maudlin I Simarro PP. Controlling sleeping sickness-a review. Parasitology (2009) 136:1943–9. 10.1017/S0031182009006416 19691861

[4] Hotez PJ Fenwick A Savioli L Molyneux DH Rescuing the bottom billion through control of neglected tropical diseases. Lancet (2009) 373:1570–5. 10.1016/S0140-6736(09)60233-6 19410718

[5] Simarro PP Diarra A Ruiz Postigo JA Franco JR Jannin JG The human African trypanosomiasis control and surveillance programme of the World Health Organization 2000-2009: the way forward. PLoS Negl Trop Dis (2011) 5:e1007 10.1371/journal.pntd.0001007 21364972 PMC3042999

[6] Odiit M Shaw A Welburn SC Fèvre EM Coleman PG McDermott JJ. Assessing the patterns of health-seeking behaviour and awareness among sleeping-sickness patients in eastern Uganda. Ann Trop Med Parasitol (2004) 98:339–48. 10.1179/000349804225003389 15228715

[7] Geiger A Ponton F Simo G Adult blood-feeding tsetse flies, trypanosomes, microbiota and the fluctuating environment in sub-Saharan Africa. ISME J (2014) 9:1496–507. 10.1038/ismej.2014.236 25500509 PMC4478693

[8] Chappuis F Lima MA Flevaud L Ritmeijer K Human African trypanosomiasis in areas without surveillance. Emerg Infect Dis (2010) 16:354–6. 10.3201/eid1602.090967 20113585 PMC2958013

[9] Berrang-Ford L Lundine J Breau S. Conflict and human African trypanosomiasis. Soc Sci Med (2011) 72:398–407. 10.1016/j.socscimed.2010.06.006 20619948

[10] Njiokou F Nimpaye H Simo G Njitchouang GR Asonganyi T Cuny G Domestic animals as potential reservoir hosts of *Trypanosoma brucei* gambiense in sleeping sickness foci in Cameroon. Parasite (2010) 17:61–6. 10.1051/parasite/2010171061 20387740

[11] Moore S Shrestha S Tomlinson KW Vuong H. Predicting the effect of climate change on African trypanosomiasis: integrating epidemiology with parasite and vector biology. J R Soc Interface (2012) 9:817–30. 10.1098/rsif.2011.0654 22072451 PMC3306657

[12] Dumas M Bouteille B Current status of trypanosomiasis. Med Trop (1997) 57:65–9. 9513181

[13] Holzmuller P Grébaut P Semballa S Gonzatti MI Geiger A. Proteomics: a new way to improve human African trypanosomiasis diagnosis? Expert Rev Proteomics (2013) 10:289–301. 10.1586/epr.13.14 23777218

[14] Jacobs RT Plattner JJ Nare B Wring SA Chen D Freund Y Benzoxaboroles: a new class of potential drugs for human African trypanosomiasis. Future Med Chem (2011) 3:1259–78. 10.4155/fmc.11.80 21859301

[15] Torreele E Bourdin Trunz B Tweats D Kaiser M Brun R Mazué G Fexinidazole – a new oral nitroimidazole drug candidate entering clinical development for the treatment of sleeping sickness. PLoS Negl Trop Dis (2010) 4:e923 10.1371/journal.pntd.0000923 21200426 PMC3006138

[16] Barrett MP. Potential new drugs for human African trypanosomiasis: some progress at last. Curr Opin Infect Dis (2010) 23:603–8. 10.1097/QCO.0b013e32833f9fd0 20844428

[17] Bacchi CJ. Chemotherapy of human african trypanosomiasis. Interdiscip Perspect Infect Dis (2009) 2009:195040. 10.1155/2009/195040 19707529 PMC2730475

[18] Welburn SC Maudlin I. Chapter 4 – Priorities for the elimination of Sleeping Sickness. In Advances in Parasitology, editors Rollinson D. Hay S.I. Oxford: Elsevier Ltd. (2012). 79:299–337. 10.1016/B978-0-12-398457-9.00004-4 22726645

[19] Teixeira SM Vieira LQ Gazzinelli RT Genomics, pathogenesis and control of infection with protozoan parasites. Trends Parasitol (2002) 18:52–4. 10.1016/S1471-4922(01)02208-5 11832286

[20] Carabarin-Lima A Gonzalez-Vazquez MC Rodriguez-Morales O Baylon-Pacheco L Rosales-Encina JL Reyes-Lopez PA Chagas disease (American trypanosomiasis) in Mexico: an update. Acta Trop (2013) 127:126–35. 10.1016/j.actatropica.2013.04.007 23643518

[21] Barrias ES de Carvalho TM De Souza W. *Trypanosoma cruzi*: entry into mammalian host cells and parasitophorous vacuole formation. Front Immunol (2013) 4:186. 10.3389/fimmu.2013.00186 23914186 PMC3730053

[22] WHO. Control of Chagas’ Disease. Technical Report Series No. 881, WHO Technical Report Series No. 905. Geneva, Switzerland: World Health Organization (2002). 113 p 12092045

[23] Junqueira C Caetano B Bartholomeu DC Melo MB Ropert C Rodrigues MM The endless race between *Trypanosoma cruzi* and host immunity: lessons for and beyond Chagas disease. Expert Rev Mol Med (2010) 15(12):e29. 10.1017/S1462399410001560 20840799

[24] WHO. (2010). Avaialble from: http://www.who.int/leishmaniasis/en/

[25] Chappuis F Sundar S Hailu A Ghalib H Rijal S Peeling RW Visceral leishmaniasis: what are the needs for diagnosis, treatment and control? Nat Rev Microbiol (2007) 5:873–82. 10.1038/nrmicro1748 17938629

[26] Gupta G Oghumu S Satoskar AR. Mechanisms of immune evasion in leishmaniasis. Adv Appl Microbiol (2013) 82:155–84. 10.1016/B978-0-12-407679-2.00005-3 23415155 PMC3697132

[27] Alvar J Velez ID Bern C Herrero M Desjeux P Cano J Leishmaniasis worldwide and global estimates of its incidence. PLoS One (2012) 7:e35671. 10.1371/journal.pone.0035671 22693548 PMC3365071

[28] Ostyn B Gidwani K Khanal B Picado A Chappuis F Singh SP Incidence of symptomatic and asymptomatic *Leishmania donovani* infections in high-endemic foci in India and Nepal: a prospective study. PLoS Negl Trop Dis (2011) 5:e1284. 10.1371/journal.pntd.0001284 21991397 PMC3186756

[29] Silveira FT Lainson R Crescente JA de Souza AA Campos MB Gomes CM A prospective study on the dynamics of the clinical and immunological evolution of human *Leishmania (L.) infantum chagasi* infection in the Brazilian Amazon region. Trans R Soc Trop Med Hyg (2010) 104:529–35. 10.1016/j.trstmh.2010.05.002 20538310

[30] Koltas IS Eroglu F Uzun S Alabaz D. A comparative analysis of different molecular targets using PCR for diagnosis of old world leishmaniasis. Exp Parasitol (2016) 164:43–8. 10.1016/j.exppara.2016.02.007 26896641

[31] Margioto Teston AP Paula de Abreu A Gruendling AP Bahia MT Gomes ML Marques de Araújo S Differential parasitological, molecular, and serological detection of *Trypanosoma cruzi* I, II, and IV in blood of experimentally infected mice. Exp Parasitol (2016) 166:44–50. 10.1016/j.exppara.2016.03.013 26995535

[32] Ribeiro JM Walker FA. High affinity histamine-binding and antihistaminic activity of the salivary nitric oxide-carrying heme protein (nitrophorin) of *Rhodnius prolixus*. J Exp Med (1994) 180:2251–7. 10.1084/jem.180.6.2251 7964498 PMC2191789

[33] Ribeiro JMC Assumpcao TCF Pham VM Fracischetti IMB Reiseman CE. An insight into the sialotranscriptome of *Triatoma rubida* (Hemiptera: Heteroptera). J Med Entomol (2012) 49:563–72. 10.1603/ME11243 22679863 PMC3544468

[34] Ribeiro JM Mans BJ Arcà B. An insight into the sialome of blood-feeding Nematocera. Insect Biochem Mol Biol (2010) 40:767–84. 10.1016/j.ibmb.2010.08.002 20728537 PMC2950210

[35] Francischetti IM Ribeiro JM Champagne D Andersen J. Purification, cloning, expression, and mechanism of action of a novel platelet aggregation inhibitorfrom the salivary gland of the blood-sucking bug, *Rhodnius prolixus*. J Biol Chem (2000) 275:12639–50. 10.1074/jbc.275.17.12639 10777556

[36] Montandon CE Barros E Vidigal PM Mendes MT Anhê AC de Oliveira Ramos HJ Comparative proteomic analysis of the saliva of the *Rhodnius prolixus*, *Triatoma lecticularia* and *Panstrongylus herreri* triatomines reveals a high interespecific functional biodiversity. Insect Biochem Mol Biol (2016) 71:83–90. 10.1016/j.ibmb.2016.02.009 26940473

[37] Ribeiro JM Genta FA Sorgine MH Logullo R Mesquita RD Paiva-Silva GO An insight into the transcriptome of the digestive tract of the bloodsucking bug, *Rhodnius prolixus*. PLoS Negl Trop Dis (2014) 8:e2594. 10.1371/journal.pntd.0002594 24416461 PMC3886914

[38] Santos A Ribeiro JMC Lehane MJ Gontijo NF Veloso AB Sant’Anna MRV The sialotranscriptome of the blood-sucking bug *Triatoma brasiliensis* (Hemiptera, Triatomine). Insect Mol Biol (2007) 37:7012–7012. 10.1016/j.ibmb.2007.04.004 PMC1896098 17550826

[39] Alves-silva J Ribeiro JMC Van den Abbeele J Attardo G Hao Z Haines LR An insight into the sialome of *Glossina morsitans morsitans*. BMC Genomics (2013) 11:213 10.1186/1471-2164-11-213 20353571 PMC2853526

[40] Cappello M Li S Chen X Li CB Harrison L Narashimhan S Tsetse thrombin inhibitor: bloodmeal-induced expression of an anticoagulant in salivary glands and gut tissue of *Glossina morsitans morsitans*. Proc Natl Acad Sci U S A (1998) 95:14290–5. 10.1073/pnas.95.24.14290 9826693 PMC24366

[41] Charlab R Valenzuela JG Rowton ED Ribeiro JMC. Toward an understanding of the biochemical and pharmacological complexity of the saliva of a hematophagous sand fly *Lutzomyia longipalpis*. Proc Natl Acad Sci U S A (1999) 96:15155–60. 10.1073/pnas.96.26.15155 10611354 PMC24789

[42] Li S Kwon J Askoy S. Characterization of genes expressed in the salivary glands of the tse tse fly, *Glossina morsitans morsitans*. Insect Mol Biol (2001) 10:69–76. 10.1046/j.1365-2583.2001.00240.x 11240638

[43] Andersen JF Ribeiro JM. A secreted salivary inositol polyphosphate 5-phosphatase from a blood-feeding insect: allosteric activation by soluble phosphoinositides and phosphatidylserine. Biochemistry (2006) 45:5450–7. 10.1021/bi052444j 16634626

[44] Morita T. Structures and functions of snake venom CLPs (C-type lectin-like proteins) with anticoagulant-, procoagulant-, and platelet-modulating activities. Toxicon (2005) 45:1099–114. 10.1016/j.toxicon.2005.02.021 15922777

[45] Noeske-Jungblut C Krätzschmar J Haendler B Alagon A Possani L Verhallen P An inhibitor of collagen-induced platelet agregation from the saliva of *Triatoma pallidipennis*. J Biol Chem (1994) 269:5050–3. 8106481

[46] Noeske-Jungblut C Haendler B Donner P Alagon A Possani L Schleuning WD. Triabin, a highly potent exosite inhibitor of thrombin. J Biol Chem (1995) 270:28629–34. 10.1074/jbc.270.48.28629 7499380

[47] Paddock CD McKerrow JH Hansell E Foreman KW Hsieh I Marshall N. Identification, cloning, and recombinant expression of procalin, a major triatomine allergen. J Immunol (2001) 167:2694–9. 10.4049/jimmunol.167.5.2694 11509613

[48] Assumpção TC Alvarenga PH Ribeiro JM Andersen JF Francischetti IM. Dipetalodipin, a novel multifunctional salivary lipocalin that inhibits platelet aggregation, vasoconstriction, and angiogenesis through unique binding specificity for TXA2, PGF2alpha, and 15(S)-HETE. J Biol Chem (2010) 285:39001–12. 10.1074/jbc.M110.152835 20889972 PMC2998087

[49] Lopes MF Zamboni DS Lujan HD Rodrigues MM Immunity to protozoan parasites. J Parasitol Res (2012) 2012:250793 10.1155/2012/250793 22619699 PMC3352617

[50] Mendes-Sousa AF Nascimento AA Queiroz DC Vale VF Fujiwara RT Araújo RN Different host complement systems and their interactions with saliva from *Lutzomyia longipalpis* (Diptera, Psychodidae) and *Leishmania infantum* promastigotes. PLoS One (2013) 8:e79787. 10.1371/journal.pone.0079787 24255715 PMC3821853

[51] Joiner KA Complement evasion by bacteria and parasites. Annu Rev Microbiol (1988) 42:201–30. 10.1146/annurev.mi.42.100188.001221 3059994

[52] Puentes SM Dwyer DM Bates PA Joiner KA. Binding and release of C3 from *Leishmania donovani* promastigotes during incubation in normal human serum. J Immunol (1989) 143:3743–9. 2584716

[53] McConville MJ Ralton JE. Developmentally regulated changes in the cell surface architecture of *Leishmania* parasites. Behring Inst Mitt (1997) 99:34–43. 9303200

[54] Brittingham A Morrison CJ McMaster WR McGwire BS Chang KP Mosser DM. Role of the *Leishmania* surface protease gp63 in complement fixation, cell adhesion, and resistance to complement-mediated lysis. J Immunol (1995) 155:3102–11. 7673725

[55] Lincoln LM Ozaki M Donelson JE Beetham JK Genetic complementation of *Leishmania* deficient in PSA (GP46) restores their resistance to lysis by complement. Mol Biochem Parasitol (2004) 137:185–9. 10.1016/j.molbiopara.2004.05.004 15279966

[56] Späth GF Epstein L Leader B Singer SM Avila HA Turco SJ Lipophosphoglycan is a virulence factor distinct from related glycoconjugates in the protozoan parasite *Leishmania major*. Proc Natl Acad Sci U S A (2000) 97:9258–63. 10.1073/pnas.160257897 10908670 PMC16855

[57] Bhandari V Kumar D Verma S Srividya G Negi NS Singh R Increased parasite surface antigen-2 expression in clinical isolates of *Leishmania donovani* augments antimony resistance. Biochem Biophys Res Commun (2013) 440:646–51. 10.1016/j.bbrc.2013.09.113 24103752

[58] Norris KA Bradt B Cooper NR So M Characterization of a *Trypanosoma cruzi* C3 binding protein with functional and genetic similarities to the human complement regulatory protein, decayaccelerating factor. J Immunol (1991) 147:2240–7. 1717552

[59] Zambrano-Villa S Rosales-Borjas D Carrero JC Ortiz-Ortiz L. How protozoan parasites evade the immune response. Trends Parasitol (2002) 18:272–8. 10.1016/S1471-4922(02)02289-4 12036742

[60] Cestari I Evans-Osses I Schlapbach LJ de Messias-Reason I Ramirez MI Mechanisms of complement lectin pathway activation and resistance by trypanosomatid parasites. Mol Immunol (2012) 53:328–34. 10.1016/j.molimm.2012.08.015 23063472

[61] Raper J Portela MP Lugli E Frevert U Tomlinson S. Trypanosome lytic factors: novel mediators of human innate immunity. Curr Opin Microbiol (2001) 4:402–8. 10.1016/S1369-5274(00)00226-5 11495802

[62] Lecordier L Uzureau P Tebabi P Pérez-Morga D Nolan D Schumann Burkard G Identification of *Trypanosoma brucei* components involved in trypanolysis by normal human serum. Mol Microbiol (2014) 94:625–36. 10.1111/mmi.12783 25256834

[63] Pays E Vanhollebeke B Uzureau P Lecordier L Pérez-Morga D. The molecular arms race between African trypanosomes and humans. Nat Rev Microbiol (2014) 12:575–84. 10.1038/nrmicro3298 24975321

[64] Capewell P Clucas C DeJesus E Kieft R Hajduk S Veitch N The TgsGP gene is essential for resistance to human serum in *Trypanosoma brucei* gambiense. PLoS Pathog (2013) 9:e1003686. 10.1371/journal.ppat.1003686 24098129 PMC3789759

[65] Burleigh BA Woolsey AM. Cell signalling and *Trypanosoma cruzi* invasion. Cell Microbiol (2002) 4:701–11. 10.1046/j.1462-5822.2002.00226.x 12427093

[66] de Souza W de Carvalho TM Barrias ES. Review on *Trypanosoma cruzi*: host cell interaction. Int J Cell Biol (2010) 2010:295394. 10.1155/2010/295394 20811486 PMC2926652

[67] Sibley LD Andrews NW. Cell invasion by un-palatable parasites. Traffic (2000) 1:100–6. 10.1034/j.1600-0854.2000.010202.x 11208090

[68] Amer AO Swanson MS. A phagosome of one’s own: a microbial guide to life in the macrophage. Curr Opin Microbiol (2002) 5:56–61. 10.1016/S1369-5274(02)00286-2 11834370

[69] Andrews NW Abrams CK Slatin SL Griffiths G. A *T. cruzi*-secreted protein immunologically related to the complement component C9: evidence for membrane pore-forming activity at low pH. Cell (1990) 61:1277–87. 10.1016/0092-8674(90)90692-8 2194668

[70] Rubin-de-Celis SS Uemura H Yoshida N Schenkman S. Expression of trypomastigote trans-sialidase in metacyclic forms of *Trypanosoma cruzi* increases parasite escape from its parasitophorous vacuole. Cell Microbiol (2006) 8:1888–98. 10.1111/j.1462-5822.2006.00755.x 16824037

[71] Burleigh BA. Host cell signaling and *Trypanosoma cruzi* invasion: do all roads lead to lysosomes? Sci STKE (2005) 2005:e36. 10.1126/stke.2932005pe36 16030288

[72] Walker DM Oghumu S Gupta G McGwire BS Drew ME Satoskar AR. Mechanisms of cellular invasion by intracellular parasites. Cell Mol Life Sci (2014) 71(7):1245–63. 10.1007/s00018-013-1491-1 24221133 PMC4107162

[73] Vannier-Santos MA Martiny A de Souza W. Cell biology of *Leishmania* spp.: invading and evading. Curr Pharm Des (2002) 8:297–318. 10.2174/1381612023396230 11860368

[74] Alexander J Russell DG The interaction of *Leishmania* species with macrophages. Adv Parasitol (1992) 31:175–254. 10.1016/S0065-308X(08)60022-6 1496927

[75] Courret N Fréhel C Gouhier N Pouchelet M Prina E Roux P Biogenesis of *Leishmania*-harbouring parasitophorous vacuoles following phagocytosis of the metacyclic promastigote or amastigote stages of the parasites. J Cell Sci (2002) 115:2303–16. 12006615 10.1242/jcs.115.11.2303

[76] Freitas-Teixeira PM Silveira-Lemos D Giunchetti RC Baratta-Masini A Mayrink W Peruhype-Magalhães V Distinct pattern of immunophenotypic features of innate and adaptive immunity as a putative signature of clinical and laboratorial status of patients with localized cutaneous leishmaniasis. Scand J Immunol (2012) 76:421–32. 10.1111/j.1365-3083.2012.02748.x 22823491

[77] Terrazas C Oghumu S Varikuti S Martinez-Saucedo D Beverley SM Satoskar AR. Uncovering *Leishmania*-macrophage interplay using imaging flow cytometry. J Immunol Methods (2015) 423:93–8. 10.1016/j.jim.2015.04.022 25967951 PMC4620550

[78] Horn D. Antigenic variation in African trypanosomes. Mol Biochem Parasitol (2014) 195:123–9. 10.1016/j.molbiopara.2014.05.001 24859277 PMC4155160

[79] Carrington M Carnall N Crow MS Gaud A Redpath MB Wasunna CL The properties and function of the glycosylphosphatidylinositol-phospholipase C in *Trypanosoma brucei*. Mol Biochem Parasitol (1998) 91:153–64. 10.1016/S0166-6851(97)00190-4 9574933

[80] Magez S Stijlemans B Radwanska M Pays E Ferguson MA De Baetselier P The glycosyl-inositol-phosphate and dimyristoylglycerol moieties of the glycosylphosphatidyl-inositol anchor of the trypanosome variant-specific surface glycoprotein are distinct macrophage-activating factors. J Immunol (1998) 160:1949–56. 9469458

[81] Paulnock DM Coller SP. Analysis of macrophage activation in African trypanosomiasis. J Leukoc Biol (2001) 69:685–90. 11358974

[82] Mansfield JM Paulnock DM. Genetic manipulation of African trypanosomes as a tool to dissect the immunobiology of infection. Immunology (2008) 30:245–53. 10.1111/j.1365-3024.2007.01003.x 18208450

[83] Namangala B. How the African trypanosomes evade host immune killing. Parasite Immunol (2011) 33:430–7. 10.1111/j.1365-3024.2011.01280.x 21261664

[84] Chaussabel D Pajak B Vercruysse V Bisseyé C Garzé V Habib M Alteration of migration and maturation of dendritic cells and T-cell depletion in the course of experimental *Trypanosoma cruzi* infection. Lab Invest (2003) 83:1373–82. 10.1097/01.LAB.0000087587.93781.6F 13679445

[85] Macauley MS Crocker PR Paulson JC. Siglec-mediated regulation of immune cell function in disease. Nat Rev Immunol (2014) 14(10):653–66. 10.1038/nri3737 25234143 PMC4191907

[86] Khatua B Roy S Mandal C. Sialic acids siglec interaction: a unique strategy to circumvent innate immune response by pathogens. Indian J Med Res (2013) 138:648–62. 24434319 PMC3928697

[87] Freire-de-Lima L Alisson-Silva F Carvalho ST Takiya CM Rodrigues MM DosReis GA *Trypanosoma cruzi* subverts host cell sialylation and may compromise antigen-specific CD8+ T cell responses. J Biol Chem (2010) 285:13388–96. 10.1074/jbc.M109.096305 20106975 PMC2859498

[88] Shrikant PA Rao R Li Q Kesterson J Eppolito C Mischo A Regulating functional cell fates in CD8 T cells. Immunol Res (2010) 46:12–22. 10.1007/s12026-009-8130-9 19859830 PMC5049497

[89] Stempin C Giordanengo L Gea S Cerbán F. Alternative activation and increase of *Trypanosoma cruzi* survival in murine macrophages stimulated by cruzipain, a parasite antigen. J Leukoc Biol (2002) 72:727–34. 12377942

[90] Nagajyothi F Machado FS Burleigh BA Jelicks LA Scherer PE Mukherjee S Mechanisms of *Trypanosoma cruzi* persistence in Chagas disease. Cell Microbiol (2012) 14:634–43. 10.1111/j.1462-5822.2012.01764.x 22309180 PMC3556388

[91] Arango Duque G Descoteaux A. *Leishmania* survival in the macrophage: where the ends justify the means. Curr Opin Microbiol (2015) 26:32–40. 10.1016/j.mib.2015.04.007 25988701

[92] Matheoud D Moradin N Bellemare-Pelletier A Shio MT Hong WJ Olivier M *Leishmania* evades host immunity by inhibiting antigen cross-presentation through direct cleavage of the SNARE VAMP8. Cell Host Microbe (2013) 14:15–25. 10.1016/j.chom.2013.06.003 23870310

[93] Gurung P Kanneganti TD. Innate immunity against *Leishmania* infections. Cell Microbiol (2015) 17:1286–94. 10.1111/cmi.12484 26249747 PMC4698274

[94] Bhardwaj N Rosas LE Lafuse WP Satoskar AR. *Leishmania* inhibits STAT1-mediated IFN-gamma signaling in macrophages: increased tyrosine phosphorylation of dominant negative STAT1beta by *Leishmania mexicana*. Int J Parasitol (2005) 35:75–82. 10.1016/j.ijpara.2004.10.018 15619518

[95] Forget G Matte C Siminovitch KA Rivest S Pouliot P Olivier M Regulation of the Leishmania-induced innate inflammatory response by the protein tyrosine phosphatase SHP-1. Eur J Immunol (2005) 35(6):1906–17. 15902687 10.1002/eji.200526037

[96] Forget G Gregory DJ Whitcombe LA Olivier M. Role of host protein tyrosine phosphatase SHP-1 in *Leishmania donovani*-induced inhibition of nitric oxide production. Infect Immun (2006) 74:6272–9. 10.1128/IAI.00853-05 17057094 PMC1695482

[97] Forget G Matte C Siminovitch KA Rivest S Pouliot P Olivier M Regulation of the *Leishmania* induced innate inflammatory response by the protein tyrosine phosphatase SHP-1. Eur J Immunol (2005) 35:1906–17. 10.1002/eji.200526037 15902687

[98] Forget G Gregory DJ Olivier M. Proteasome-mediated degradation of STAT1alpha following infection of macrophages with *Leishmania donovani*. J Biol Chem (2005) 280:30542–9. 10.1074/jbc.M414126200 15983048

[99] Cecílio P Pérez-Cabezas B Santarém N Maciel J Rodrigues V Cordeiro da Silva A. Deception and manipulation: the arms of *Leishmania*, a successful parasite. Front Immunol (2014) 5:480. 10.3389/fimmu.2014.00480 25368612 PMC4202772

[100] Vincendeau P Gobert AP Daulouède S Moynet D Mossalayi MD Arginases in parasitic diseases. Trends Parasitol (2003) 19:9–12. 10.1016/S1471-4922(02)00010-7 12488215

[101] Gobert AP Daulouede S Lepoivre M Boucher JL Bouteille B Buguet A l-Arginine availability modulates local nitric oxide production and parasite killing in experimental trypanosomiasis. Infect Immun (2000) 68:4653–7. 10.1128/IAI.68.8.4653-4657.2000 10899869 PMC98402

[102] De Muylder G Daulouède S Lecordier L Uzureau P Morias Y Van Den Abbeele J A *Trypanosoma brucei* kinesin heavy chain promotes parasite growth by triggering host arginase activity. PLoS Pathog (2013) 9:e1003731. 10.1371/journal.ppat.1003731 24204274 PMC3814429

[103] Taheri F Ochoa JB Faghiri Z Culotta K Park HJ Lan MS l-Arginine regulates the expression of the T-cell receptor zeta chain (CD3zeta) in Jurkat cells. Clin Cancer Res (2001) 7:958s–65s. 11300497

[104] Daulouède S Bouteille B Moynet D De Baetselier P Courtois P Lemesre JL Human macrophage tumor necrosis factor (TNF)-alpha production induced by *Trypanosoma brucei* gambiense and the role of TNF-alpha in parasite control. J Infect Dis (2001) 183:988–91. 10.1086/319257 11237819

[105] Salmon D Vanwalleghem G Morias Y Denoeud J Krumbholz C Lhommé F Adenylate cyclases of *Trypanosoma brucei* inhibit the innate immune response of the host. Science (2012) 337:463–6. 10.1126/science.1222753 22700656

[106] Evans-Osses I de Messias-Reason I Ramirez MI. The emerging role of complement lectin pathway in trypanosomatids: molecular bases in activation, genetic deficiencies, susceptibility to infection, and complement system-based therapeutics. ScientificWorldJournal (2013) 2013:675898. 10.1155/2013/675898 23533355 PMC3595680

[107] Evans-Osses I Mojoli A Beltrame MH da Costa DE DaRocha WD Velavan TP Differential ability to resist to complement lysis and invade host cells mediated by MBL in R4 and 860 strains of *Trypanosoma cruzi*. FEBS Lett (2014) 588:956–61. 10.1016/j.febslet.2014.01.054 24560788

[108] Ribeiro CH Lynch NJ Stover CM Ali YM Valck C Noya-Leal F Deficiency in mannose-binding lectin-associated serine protease-2 does not increase susceptibility to *Trypanosoma cruzi* infection. Am J Trop Med Hyg (2015) 92:320–4. 10.4269/ajtmh.14-0236 25548381 PMC4347335

[109] Ramirez G Valck C Ferreira VP López N Ferreira A Extracellular *Trypanosoma cruzi* calreticulin in the host-parasite interplay. Trends Parasitol (2012) 27:115–22. 10.1016/j.pt.2010.12.007 21288773

[110] Sánchez-Valdéz FJ Pérez Brandán C Ramírez G Uncos AD Zago MP Cimino RO A monoallelic deletion of the TcCRT gene increases the attenuation of a cultured *Trypanosoma cruzi* strain, protecting against an in vivo virulent challenge. PLoS Negl Trop Dis (2014) 8:e2696. 10.1371/journal.pntd.0002696 24551259 PMC3923724

[111] Castillo C Ramírez G Valck C Aguilar L Maldonado I Rosas C The interaction of classical complement component C1 with parasite and host calreticulin mediates *Trypanosoma cruzi* infection of human placenta. PLoS Negl Trop Dis (2013) 7:e2376. 10.1371/journal.pntd.0002376 23991234 PMC3749977

[112] Polando R Dixit UG Carter CR Jones B Whitcomb JP Ballhorn W The roles of complement receptor 3 and Fcγ receptors during *Leishmania* phagosome maturation. J Leukoc Biol (2013) 93(6):921–32. 10.1189/jlb.0212086 23543768 PMC3656333

[113] Ricardo-Carter C Favila M Polando RE Cotton RN Bogard Horner K Condon D *Leishmania major* inhibits IL-12 in macrophages by signalling through CR3 (CD11b/CD18) and down-regulation of ETS-mediated transcription. Parasite Immunol (2013) 35:409–20. 10.1111/pim.12049 23834512 PMC4001918

[114] Green PJ Feizi T Stoll MS Thiel S Prescott A McConville MJ. Recognition of the major cell surface glycoconjugates of *Leishmania* parasites by the human serum mannan-binding protein. Mol Biochem Parasitol (1994) 66:319–28. 10.1016/0166-6851(94)90158-9 7808481

[115] Ambrosio AR De Messias-Reason IJ. *Leishmania (Viannia) braziliensis*: interaction of mannose-binding lectin with surface glycoconjugates and complement activation. An antibody-independent defence mechanism. Parasite Immunol (2005) 27:333–40. 10.1111/j.1365-3024.2005.00782.x 16149991

[116] Wilson ME Pearson RD. Roles of CR3 and mannose receptors in the attachment and ingestion of *Leishmania donovani* by human mononuclear phagocytes. Infect Immun (1988) 56:363–9. 2962944 10.1128/iai.56.2.363-369.1988 PMC259289

[117] Liu D Uzonna JE The early interaction of *Leishmania* with macrophages and dendritic cells and its influence on the host immune response. Front Cell Infect Microbiol (2012) 2:83 10.3389/fcimb.2012.00083 22919674 PMC3417671

[118] Bosetto MC Giorgio S. *Leishmania amazonensis*: multiple receptor-ligand interactions are involved in amastigote infection of human dendritic cells. Exp Parasitol (2007) 116:306–10. 10.1016/j.exppara.2007.01.003 17320869

[119] Laufs H Müller K Fleischer J Reiling N Jahnke N Jensenius JC Intracellular survival of *Leishmania major* in neutrophil granulocytes after uptake in the absence of heat-labile serum factors. Infect Immun (2002) 70:826–35. 10.1128/IAI.70.2.826-835.2002 11796617 PMC127667

[120] Peters C Kawakami M Kaul M Ilg T Overath P Aebischer T. Secreted proteophosphoglycan of *Leishmania mexicana* amastigotes activates complement by triggering the mannan binding lectin pathway. Eur J Immunol (1997) 27:2666–72. 10.1002/eji.1830271028 9368624

[121] Dagenais TR Freeman BE Demick KP Paulnock DM Mansfield JM. Processing and presentation of variant surface glycoprotein molecules to T cells in African trypanosomiasis. J Immunol (2009) 183:3344–55. 10.4049/jimmunol.0802005 19675169 PMC2730433

[122] Vincendeau P Bouteille B. Immunology and immunopathology of African trypanosomiasis. An Acad Bras Cienc (2006) 78:645–65. 10.1590/S0001-37652006000400004 17143404

[123] Flávia Nardy A Freire-de-Lima CG Morrot A. Immune evasion strategies of *Trypanosoma cruzi*. J Immunol Res (2015) 2015:178947. 10.1155/2015/178947 26240832 PMC4512591

[124] Bogdan C Röllinghoff M Solbach W. Evasion strategies of *Leishmania* parasites. Parasitol Today (1990) 6:183–7. 10.1016/0169-4758(90)90350-D 15463335

[125] Hudson KM Byner C Freeman J Terry RJ Immunodepression, high IgM levels and evasion of the immune response in murine trypanosomiasis. Nature (1976) 264:256–8. 10.1038/264256a0 1087372

[126] Sharpe RT Langley AM Mowat GN Macaskill JA Holmes PH. Immunosuppression in bovine trypanosomiasis: response of cattle infected with *Trypanosoma congolense* to foot-and-mouth disease vaccination and subsequent live virus challenge. Res Vet Sci (1982) 32:289–93. 6285433

[127] Namangala B de Baetselier P Brijs L Stijlemans B Noël W Pays E Attenuation of *Trypanosoma brucei* is associated with reduced immunosuppression and concomitant production of Th2 lymphokines. J Infect Dis (2000) 181:1110–20. 10.1086/315322 10720538

[128] Gomez-Rodriquez J Stijlemans B De Muylder G Korf H Brys L Berberof M Identification of a parasitic modulatory protein triggering the development of suppressive M1 macrophages during African trypanosomiasis. J Infect Dis (2009) 200:1849–60. 10.1086/648374 19911988

[129] Stijlemans B Vankrunkelsven A Brys L Raes G Magez S De Baetselier P. Scrutinizing the mechanisms underlying the induction of anemia of inflammation through GPI-mediated modulation of macrophage activation in a model of African trypanosomiasis. Microbes Infect (2010) 12:389–99. 10.1016/j.micinf.2010.02.006 20197106

[130] Munder M Eichmann K Morán JM Centeno F Soler G Modolell M Th1/Th2-regulated expression of arginase isoforms in murine macrophages and dendritic cells. J Immunol (1999) 163:3771–7. 10490974

[131] Garzón E Holzmuller P Bras-Gonçalves R Vincendeau P Cuny G Lemesre JL The *Trypanosoma brucei* gambiense secretome impairs lipopolysaccharide-induced maturation, cytokine production, and allostimulatory capacity of dendritic cells. Infect Immun (2013) 81:3300–8. 10.1128/IAI.00125-13 23798533 PMC3754197

[132] Namangala B Brys L Magez S De Baetselier P Beschin A *Trypanosoma brucei* brucei infection impairs MHC class II antigen presentation capacity of macrophages. Parasite Immunol (2000) 22:361–70. 10.1046/j.1365-3024.2000.00314.x 10886720

[133] De Baetselier P T-cell subsets and cytokine interplay in infectious disease. In: Mustafa AS Al-Attiyah RJ Nath I Chugh TD, editors. Mechanisms Underlying Trypanosome Induced T-Cell Immunosuppression. Basle: S. Karger (1996). p. 124–39.

[134] Noel W Hassanzadeh G Raes G Namangala B Daems I Brys L Infection stage-dependent modulation of macrophage activation in *Trypanosoma congolense*-resistant and -susceptible mice. Infect Immun (2002) 70:6180–7. 10.1128/IAI.70.11.6180-6187.2002 12379696 PMC130440

[135] Taylor KA. Immune responses of cattle to African trypanosomes: protective or pathogenic? Int J Parasitol (1998) 28:219–40. 10.1016/S0020-7519(97)00154-9 9512986

[136] Hertz CJ Filutowicz H Mansfield JM Resistance to African tyrpanosomes is IFN-c dependent. J Immunol (1998) 161:6775–83. 9862708

[137] Norris KA Schrimpf JE Szabo MJ. Identification of the gene family encoding the 160-kilodalton *Trypanosoma cruzi* complement regulatory protein. Infect Immun (1997) 65:349–57. 9009282 10.1128/iai.65.2.349-357.1997 PMC174602

[138] Samudio M Montenegro-James S Cabral M Martinez J Rojas De Arias A James MA. Cytokine responses in *Trypanosoma cruzi*-infected children in Paraguay. Am J Trop Med Hyg (1998) 58:119–21. 9452302 10.4269/ajtmh.1998.58.119

[139] Silva JS Morrissey PJ Grabstein KH Moller KM Anderson D Reed SG Interleukin 10 and interferon-c regulation of experimental *Trypanosoma cruzi* infection. J Exp Med (1992) 175:169–74. 10.1084/jem.175.1.169 1730915 PMC2119081

[140] Silva JS Vespa GNR Cardoso MAG Aliberti JCS Cunha FQ. Tumor necrosis factor alpha mediates resistance to *Trypanosoma cruzi* infection in mice by inducing nitric oxide production in infected gamma interferon-activated macrophages. Infect Immun (1995) 63:4862–7. 7591147 10.1128/iai.63.12.4862-4867.1995 PMC173696

[141] Cardillo F Voltarelli CJ Reed SG Silva JS. Regulation of *Trypanosoma cruzi* infection in mice by gamma interferon and interleukin 10: role of NK cells. Infect Immun (1996) 64:128–34. 8557330 10.1128/iai.64.1.128-134.1996 PMC173737

[142] Zhang L Tarleton RL Characterization of cytokine production in murine *T. cruzi* infection by in situ immunochemistry: lack of association between susceptibility and T helper type 2 cytokine production. Eur J Immunol (1996) 26:102–9. 10.1002/eji.1830260116 8566051

[143] Wenzel UA Bank E Florian C Förster S Zimara N Steinacker J *Leishmania major* parasite stage-dependent host cell invasion and immune evasion. FASEB J (2012) 26:29–39. 10.1096/fj.11-184895 21908716

[144] Buchmüller-Rouiller Y Mauël J. Impairment of the oxidative metabolism of mouse peritoneal macrophages by intracellular *Leishmania* spp. Infect Immun (1987) 55:587–93. 3546131 10.1128/iai.55.3.587-593.1987 PMC260378

[145] Bogdan C Gessner A Solbach W Röllinghoff M. Invasion, control and persistence of *Leishmania* parasites. Curr Opin Immunol (1996) 8(4):517–25. 10.1016/S0952-7915(96)80040-9 8794010

[146] Katakura K Kobayashi A. Acid phosphatase activity of virulent and avirulent clones of *Leishmania donovani* promastigotes. Infect Immun (1988) 56:2856–60. 3169990 10.1128/iai.56.11.2856-2860.1988 PMC259661

[147] McNeely TB Rosen G Londner MV Turco SJ. Inhibitory effects on protein kinase C activity by lipophosphoglycan fragments and glycosylphosphatidylinositol antigens of the protozoan parasite *Leishmania*. Biochem J (1989) 259:601–4. 10.1042/bj2590601 2524191 PMC1138552

[148] Pearson RD Harcus JL Roberts D Donowitz GR. Differential survival of *Leishmania donovani* amastigotes in human monocytes. J Immunol (1983) 131:1994–9. 6619546

[149] Handman E Schnur LF Spithill TW Mitchell GF. Passive transfer of *Leishmania* lipopolysaccharide confers parasite survival in macrophages. J Immunol (1986) 137:3608–13. 3782793

[150] Kaye P Scott P. Leishmaniasis: complexity at the host-pathogen interface. Nat Rev Microbiol (2011) 9(8):604–15. 10.1038/nrmicro2608 21747391

[151] Kaye PM Costimulation and the regulation of antimicrobial immunity. Immunol Today (1995) 16:423427 10.1016/0167-5699(95)80018-2 7546205

[152] Reiner NE. Altered cell signaling and mononuclear phagocyte deactivation during intracellular infection. Immunol Today (1994) 15:374–81. 10.1016/0167-5699(94)90176-7 7916951

[153] de Souza Leao S Lang T Prina E Hellio R Antoine JC. Intracellular *Leishmania amazonensis* amastigotes internalize and degrade MHC class II molecules of their host cells. J Cell Sci (1995) 108:3219–31. 7593283 10.1242/jcs.108.10.3219

[154] Saha B Das G Vohra H Ganguly NK Mishra GC Macrophage-T cell interaction in experimental visceral leishmaniasis: failure to express costimulatory molecules on *Leishmania*-infected macrophages and its implications in the suppression of cell-mediated immunity. Eur J Immunol (1995) 25:2492–8. 10.1002/eji.1830250913 7589116

[155] Carrada G Cañeda C Salaiza N Delgado J Ruiz A Sanchez B Monocyte cytokine and costimulatory molecule expression in patients infected with *Leishmania mexicana*. Parasite Immunol (2007) 29:117–26. 10.1111/j.1365-3024.2006.00924.x 17266739

[156] Mosmann TR Coffman RL TH1 and TH2 cells: different patterns of lymphokine secretion lead to different functional properties. Annu Rev Immunol (1989) 7:145–73. 10.1146/annurev.iy.07.040189.001045 2523712

[157] Dong L Uzonna JE. The early interaction of *Leishmania* with macrophages and dendritic cells and its influence on the host immune response. Front Cell Infect Microbiol (2012) 2:83. 10.3389/fcimb.2012.00083 22919674 PMC3417671

[158] Desjardins M Descoteaux A. Survival strategies of *Leishmania donovani* in mammalian host macrophages. Res Immunol (1998) 149:689–92. 10.1016/S0923-2494(99)80040-6 9851525

[159] Kane MM Mosser DM. The role of IL-10 in promoting disease progression in leishmaniasis. J Immunol (2001) 166:1141–7. 10.4049/jimmunol.166.2.1141 11145695

[160] León B López-Bravo M Ardavín C. Monocyte-derived dendritic cells formed at the infection site control the induction of protective T helper 1 responses against *Leishmania*. Immunity (2007) 26:519–31. 10.1016/j.immuni.2007.01.017 17412618

[161] Von Stebut E Belkaid Y Jakob T Sacks DL Udey MC. Uptake of *Leishmania major* amastigotes results in activation and interleukin 12 release from murine skin-derived dendritic cells: implications for the initiation of anti-*Leishmania* immunity. J Exp Med (1998) 188:1547–52. 10.1084/jem.188.8.1547 9782133 PMC2213412

[162] Kima PE Soong L Chicharro C Ruddle NH McMahon-Pratt D. *Leishmania*-infected macrophages sequester endogenously synthesized parasite antigens from presentation to CD4+ T cells. Eur J Immunol (1996) 26:3163–9. 10.1002/eji.1830261249 8977318

[163] Ritter U Meissner A Scheidig C Körner H. CD8 alpha- and Langerin-negative dendritic cells, but not Langerhans cells, act as principal antigen-presenting cells in leishmaniasis. Eur J Immunol (2004) 34:1542–50. 10.1002/eji.200324586 15162423

[164] Kautz-Neu K Noordegraaf M Dinges S Bennett CL John D Clausen BE Langerhans cells are negative regulators of the anti-*Leishmania* response. J Exp Med (2011) 208:885–91. 10.1084/jem.20102318 21536741 PMC3092359

[165] Costa DL Guimarães LH Cardoso TM Queiroz A Lago E Roselino AM Characterization of regulatory T cell (Treg) function in patients infected with *Leishmania braziliensis*. Hum Immunol (2013) 74(12):1491–500. 10.1016/j.humimm.2013.08.269 23993989 PMC3846617

[166] Rai AK Thakur CP Kumar P Mitra DK. Impaired expression of CD26 compromises T-cell recruitment in human visceral leishmaniasis. Eur J Immunol (2012) 42(10):2782–91. 10.1002/eji.201141912 22806538

[167] Sibley LD. Invasion and intracellular survival by protozoan parasites. Immunol Rev (2011) 240:72–91. 10.1111/j.1600-065X.2010.00990.x 21349087 PMC3697736

[168] DosReis GA. Susceptible hosts: a resort for parasites right in the eye of the immune response. An Acad Bras Cienc (2000) 72:79–82. 10.1590/S0001-37652000000100011 10932108

[169] Beschin A Van Den Abbeele J De Baetselier P Pays E. African trypanosome control in the insect vector and mammalian host. Trends Parasitol (2014) 30:538–47. 10.1016/j.pt.2014.08.006 25246021

[170] Feig C Peter ME. How apoptosis got the immune system in shape. Eur J Immunol (2007) 37(Suppl 1):S61–70. 10.1002/eji.200737462 17972347

[171] Gannavaram S Debrabant A. Programmed cell death in *Leishmania*: biochemical evidence and role in parasite infectivity. Front Cell Infect Microbiol (2012) 2:95. 10.3389/fcimb.2012.00095 22919685 PMC3417670

[172] Burleigh BA Andrews NW. Signaling and host cell invasion by *Trypanosoma cruzi*. Curr Opin Microbiol (1998) 1:461–5. 10.1016/S1369-5274(98)80066-0 10066513

[173] Aga E Katschinski DM van Zandbergen G Laufs H Hansen B Müller K Inhibition of the spontaneous apoptosis of neutrophil granulocytes by the intracellular parasite *Leishmania major*. J Immunol (2002) 169:898–905. 10.4049/jimmunol.169.2.898 12097394

[174] Ribeiro-Gomes FL Peters NC Debrabant A Sacks DL. Efficient capture of infected neutrophils by dendritic cells in the skin inhibits the early anti-*Leishmania* response. PLoS Pathog (2012) 8:e1002536. 10.1371/journal.ppat.1002536 22359507 PMC3280984

[175] Ribeiro-Gomes FL Otero AC Gomes NA Moniz-De-Souza MC Cysne-Finkelstein L Arnholdt AC Macrophage interactions with neutrophils regulate *Leishmania major* infection. J Immunol (2004) 172:4454–62. 10.4049/jimmunol.172.7.4454 15034061

[176] Peters NC Egen JG Secundino N Debrabant A Kimblin N Kamhawi S In vivo imaging reveals an essential role for neutrophils in leishmaniasis transmitted by sand flies. Science (2008) 321:970–4. 10.1126/science.1159194 18703742 PMC2606057

[177] Van Zandbergen G Klinger M Mueller A Dannenberg S Gebert A Solbach W Cutting edge: neutrophil granulocyte serves as a vector for *Leishmania* entry into macrophages. J Immunol (2004) 173:6521–5. 10.4049/jimmunol.173.11.6521 15557140

[178] Afonso L Borges VM Cruz H Ribeiro-Gomes FL DosReis GA Dutra AN Interactions with apoptotic but not with necrotic neutrophils increase parasite burden in human macrophages infected with *Leishmania amazonensis*. J Leukoc Biol (2008) 84:389–96. 10.1189/jlb.0108018 18483206

[179] Allenbach C Zufferey C Perez C Launois P Mueller C Tacchini-Cottier F. Macrophages induce neutrophil apoptosis through membrane TNF, a process amplified by *Leishmania major*. J Immunol (2006) 176:6656–64. 10.4049/jimmunol.176.11.6656 16709824

[180] Kilpatrick LE Sun S Mackie D Baik F Li H Korchak HM. Regulation of TNF mediated antiapoptotic signaling in human neutrophils: role of delta-PKC and ERK1/2. J Leukoc Biol (2006) 80:1512–21. 10.1189/jlb.0406284 17138860

[181] Moore KJ Matlashewski G. Intracellular infection by *Leishmania donovani* inhibits macrophage apoptosis. J Immunol (1994) 152:2930–7. 8144893

[182] Duleu S Vincendeau P Courtois P Semballa S Lagroye I Daulouède S Mouse strain susceptibility to trypanosome infection: an arginase-dependent effect. J Immunol (2004) 172:6298–303. 10.4049/jimmunol.172.10.6298 15128819

[183] Krammer PH Arnold R Lavrik IN. Life and death in peripheral T cells. Nat Rev Immunol (2007) 7:532–42. 10.1038/nri2115 17589543

[184] Guillermo LV Silva EM Ribeiro-Gomes FL De Meis J Pereira WF Yagita H The Fas death pathway controls coordinated expansions of type 1 CD8 and type 2 CD4 T cells in *Trypanosoma cruzi* infection. J Leukoc Biol (2007) 81:942–51. 10.1189/jlb.1006643 17261545

[185] Bertho AL Santiago MA Da-Cruz AM Coutinho SG. Detection of early apoptosis and cell death in T CD4+ and CD8+ cells from lesions of patients with localized cutaneous leishmaniasis. Braz J Med Biol Res (2000) 33:317–25. 10.1590/S0100-879X2000000300010 10719384

[186] De Lima VM Fattori KR de Souza F Eugênio FR dos Santos PS Rozza DB Apoptosis in T lymphocytes from spleen tissue and peripheral blood of *L. (L.) chagasi* naturally infected dogs. Vet Parasitol (2012) 184:147–53. 10.1016/j.vetpar.2011.08.024 21899954

[187] Potestio M D’Agostino P Romano GC Milano S Ferlazzo V Aquino A CD4+ CCR5+ and CD4+CCR3+ lymphocyte subset and monocyte apoptosis in patients with acute visceral leishmaniasis. Immunology (2004) 113:260–8. 10.1111/j.1365-2567.2004.01948.x 15379987 PMC1782561

[188] Reckling S Divanovic S Karp CL Wojciechowski S Belkaid Y Hildeman D. Proapoptotic Bcl-2 family member bim promotes persistent infection and limits protective immunity. Infect Immun (2008) 76:1179–85. 10.1128/IAI.01093-06 18086806 PMC2258821

[189] Mukherjee P Sen PC Ghose AC. Lymph node cells from BALB/c mice with chronic visceral leishmaniasis exhibiting cellular anergy and apoptosis: involvement of Ser/Thr phosphatase. Apoptosis (2006) 11:2013–29. 10.1007/s10495-006-0088-7 17013755

[190] Antoine-Moussiaux N Cornet A Cornet F Glineur S Dermine M Desmecht D. A non-cytosolic protein of *Trypanosoma evansi* induces CD45-dependent lymphocyte death. PLoS One (2009) 4:e5728. 10.1371/journal.pone.0005728 19478957 PMC2685979

[191] Bockstal V Guirnalda P Caljon G Goenka R Telfer JC Frenkel D *T. brucei* infection reduces B lymphopoiesis in bone marrow and truncates compensatory splenic lymphopoiesis through transitional B-cell apoptosis. PLoS Pathog (2011) 7:e1002089. 10.1371/journal.ppat.1002089 21738467 PMC3128123

[192] Radwanska M Guirnalda P De Trez C Ryffel B Black S Magez S Trypanosomiasis-induced B cell apoptosis results in loss of protective anti-parasite antibody responses and abolishment of vaccine-induced memory responses. PLoS Pathog (2008) 4(5):e1000078 10.1371/journal.ppat.1000078 18516300 PMC2386555

[193] Zuñiga E Motran CC Montes CL Yagita H Gruppi A. *Trypanosoma cruzi* infection selectively renders parasite-specific IgG+ B lymphocytes susceptible to Fas/Fas ligand-mediated fratricide. J Immunol (2002) 168:3965–73. 10.4049/jimmunol.168.8.3965 11937553

[194] Zuniga E Acosta-Rodriguez E Merino MC Montes C Gruppi A. Depletion of immature B cells during *Trypanosoma cruzi* infection: involvement of myeloid cells and the cyclooxygenase pathway. Eur J Immunol (2005) 35:1849–58. 10.1002/eji.200526005 15864778

[195] Vickerman K Antigenic variation in trypanosomes. Nature (1978) 273:613–7. 10.1038/273613a0 661969

[196] Depledge DP MacLean LM Hodgkinson MR Smith BA Jackson AP Ma S *Leishmania*-specific surface antigens show sub-genus sequence variation and immune recognition. PLoS Negl Trop Dis (2010) 4:e829. 10.1371/journal.pntd.0000829 20927190 PMC2946902

[197] Devera R Fernandes O Coura JR. Should *Trypanosoma cruzi* be called “cruzi” complex? A review of the parasite diversity and the potential of selecting population after in vitro culturing and mice infection. Mem Inst Oswaldo Cruz (2003) 98:1–12. 10.1590/S0074-02762003000100001 12700855

[198] Reiner NE Ng W McMaster WR. Parasite-accessory cell interactions in murine leishmaniasis. II. *Leishmania donovani* suppresses macrophage expression of class I and class II major histocompatibility complex gene products. J Immunol (1987) 138:1926–32. 2434567

[199] Flora R Aghazadeh-Dibavar S Bandyopadhyay M Dasgupta S. Immunosuppression during *Leishmania donovani* infection: a potential target for the development of therapy. Ann Parasitol (2014) 60:239–45. 25706420

[200] Tzelepis F de Alencar BC Penido ML Claser C Machado AV Bruna-Romero O Infection with *Trypanosoma cruzi* restricts the repertoire of parasite-specific CD8+ T cells leading to immunodominance. J Immunol (2008) 180:1737–48. 10.4049/jimmunol.180.3.1737 18209071

[201] Paulnock DM Smith C Mansfield JM Antigen Presenting Cell Function in African Trypanosomiasis. New York: Alan R Liss Inc (1989). p. 135–44.

[202] Shi MQ Wei GJ Tabel H. *Trypanosoma congolense* infections: MHC class II-restricted immune responses mediate either protection or disease, depending on IL-10 function. Parasite Immunol (2007) 29:107–11. 10.1111/j.1365-3024.2006.00925.x 17241399

[203] Rodrigues V Cordeiro-da-Silva A Laforge M Ouaissi A Akharid K Silvestre R Impairment of T cell function in parasitic infections. PLoS Negl Trop Dis (2014) 8:e2567. 10.1371/journal.pntd.0002567 24551250 PMC3923671

[204] Gigley JP Bhadra R Moretto MM Khan IA. T cell exhaustion in protozoan disease. Trends Parasitol (2012) 28:377–84. 10.1016/j.pt.2012.07.001 22832368 PMC3768288

[205] Dominguez MR Silveira EL de Vasconcelos JR de Alencar BC Machado AV Bruna-Romero O Subdominant/cryptic CD8 T cell epitopes contribute to resistance against experimental infection with a human protozoan parasite. PLoS One (2011) 6:e22011. 10.1371/journal.pone.0022011 21779365 PMC3136500

[206] Johnson PL Kochin BF Ahmed R Antia R. How do antigenically varying pathogens avoid cross-reactive responses to invariant antigens? Proc Biol Sci (2012) 279:2777–85. 10.1098/rspb.2012.0005 22438498 PMC3367775

[207] Onah DN Hopkins J Luckins AG. Changes in peripheral blood lymphocyte subpopulations and parasite-specific antibody responses in *Trypanosoma evansi* infection of sheep. Parasitol Res (1999) 85:263–9. 10.1007/s004360050545 10099005

[208] Mougneau E Bihl F Glaichenhaus N. Cell biology and immunology of *Leishmania*. Immunol Rev (2011) 240:286–96. 10.1111/j.1600-065X.2010.00983.x 21349100

[209] Da Silva Santos C Brodskyn CI. The role of CD4 and CD8 T cells in human cutaneous leishmaniasis. Front Public Health (2014) 2:165. 10.3389/fpubh.2014.00165 25325049 PMC4178373

[210] Sacks D Anderson C. Re-examination of the immunosuppressive mechanisms mediating non-cure of *Leishmania* infection in mice. Immunol Rev (2004) 201:225–38. 10.1111/j.0105-2896.2004.00185.x 15361244

[211] Wu G Morris J Arginine metabolism: nitric oxide and beyond. Biochem J (1998) 336:1–17. 10.1042/bj3360001 9806879 PMC1219836

[212] Lott K Zhu L Fisk JC Tomasello DL Read LK. Functional interplay between protein arginine methyltransferases in *Trypanosoma brucei*. Microbiologyopen (2014) 3:595–609. 10.1002/mbo3.191 25044453 PMC4234254

[213] Ferreira TR Alves-Ferreira EV Defina TP Walrad P Papadopoulou B Cruz AK. Altered expression of an RBP-associated arginine methyltransferase 7 in *Leishmania major* affects parasite infection. Mol Microbiol (2014) 94(5):1085–1102. 10.1111/mmi.12819 25294169

[214] Miranda MR Sayé M Bouvier LA Cámara Mde L Montserrat J Pereira CA. Cationic amino acid uptake constitutes a metabolic regulation mechanism and occurs in the flagellar pocket of *Trypanosoma cruzi*. PLoS One (2012) 7:e32760. 10.1371/journal.pone.0032760 22393446 PMC3290608

[215] Prati F Goldman-Pinkovich A Lizzi F Belluti F Koren R Zilberstein D Quinone-amino acid conjugates targeting *Leishmania* amino acid transporters. PLoS One (2014) 9:e107994. 10.1371/journal.pone.0107994 25254495 PMC4177859

[216] Da Silva MF Brodskyn LM Arginase in *Leishmania*. Subcell Biochem (2014) 74:103–17. 10.1007/978-94-007-7305-9_4 24264242

[217] Genestra M Cysne-Finkelstein L Guedes-Silva D Leon LL. Effect of l-arginine analogs and a calcium chelator on nitric oxide (NO) production by *Leishmania* sp. J Enzyme Inhib Med Chem (2003) 18:445–52. 10.1080/1475636031000138787 14692512

[218] Docampo R Moreno SN. Acidocalcisome: a novel Ca2+ storage compartment in trypanosomatids and apicomplexan parasites. Parasitol Today (1999) 15:443–8. 10.1016/S0169-4758(99)01531-8 10511686

[219] Rodríguez A Rioult MG Ora A Andrews NW. A trypanosome-soluble factor induces IP3 formation, intracellular Ca2+ mobilization and microfilament rearrangement in host cells. J Cell Biol (1995) 129:1263–73. 10.1083/jcb.129.5.1263 7775573 PMC2120476

[220] Docampo R Huang G. Calcium signaling in trypanosomatid parasites. Cell Calcium (2015) 57:194–202. 10.1016/j.ceca.2014.10.015 25468729 PMC4355036

[221] Moreno SN Docampo R. The role of acidocalcisomes in parasitic protists. J Eukaryot Microbiol (2009) 56:208–13. 10.1111/j.1550-7408.2009.00404.x 19527347 PMC2802266

[222] Eintracht J Maathai R Mellors A Ruben L. Calcium entry in *Trypanosoma brucei* is regulated by phospholipase A2 and arachidonic acid. Biochem J (1998) 336:659–66. 10.1042/bj3360659 9841878 PMC1219917

[223] Catisti R Uyemura SA Docampo R Vercesi AE. Calcium mobilization by arachidonic acid in trypanosomatids. Mol Biochem Parasitol (2000) 105:261–71. 10.1016/S0166-6851(99)00186-3 10693748

[224] Ulrich PN Lander N Kurup SP Reiss L Brewer J Soares Medeiros LC The acidocalcisome vacuolar transporter chaperone 4 catalyzes the synthesis of polyphosphate in insect-stages of *Trypanosoma brucei* and *T. cruzi*. J Eukaryot Microbiol (2014) 61:155–65. 10.1111/jeu.12093 24386955 PMC3959231

[225] King-Keller S Moore CA Docampo R Moreno SN. Ca2+ regulation of *Trypanosoma brucei* phosphoinositide phospholipase C. Eukaryot Cell (2015) 14:486–94. 10.1128/EC.00019-15 25769297 PMC4421009

[226] Benaim G Bermudez R Urbina JA. Ca2+ transport in isolated mitochondrial vesicles from *Leishmania braziliensis* promastigotes. Mol Biochem Parasitol (1990) 39:61–8. 10.1016/0166-6851(90)90008-A 2304488

[227] Benaim B Garcia CR. Targeting calcium homeostasis as the therapy of Chagas’ disease and leishmaniasis – a review. Trop Biomed (2011) 28:471–81. 22433874

[228] Roy N Chakraborty S Paul Chowdhury B Banerjee S Halder K Majumder S Regulation of PKC mediated signaling by calcium during visceral leishmaniasis. PLoS One (2014) 9(10):e110843. 10.1371/journal.pone.0110843 25329062 PMC4201563

[229] Prole DL Taylor CW. Identification of intracellular and plasma membrane calcium channel homologues in pathogenic parasites. PLoS One (2011) 6:e26218. 10.1371/journal.pone.0026218 22022573 PMC3194816

[230] Karmakar S Ukil A Mukherjee S Das PK. Regulation of guanylyl cyclase by intracellular Ca2+ in relation to the infectivity of the protozoan parasite, *Leishmania donovani*. Int J Biochem Cell Biol (2006) 38:1277–89. 10.1016/j.biocel.2006.01.002 16507348

[231] Gupta S Raychaudhury B Banerjee S Das B Datta SC. An intracellular calcium store is present in *Leishmania donovani* glycosomes. Exp Parasitol (2006) 113:161–7. 10.1016/j.exppara.2005.12.020 16513112

[232] Dolai S Pal S Yadav RK Adak S. Endoplasmic reticulum stress-induced apoptosis in *Leishmania* through Ca2+-dependent and caspase-independent mechanism. J Biol Chem (2011) 286:13638–46. 10.1074/jbc.M110.201889 21330370 PMC3075708

[233] Reimão JQ Tempone AG. Investigation into in vitro anti-leishmanial combinations of calcium channel blockers and current anti-leishmanial drugs. Mem Inst Oswaldo Cruz (2011) 106:1032–8. 10.1590/S0074-02762011000800022 22241129

[234] Marques-da-Silva C Chaves MM Chaves SP Figliuolo VR Meyer-Fernandes JR Corte-Real S Infection with *Leishmania amazonensis* upregulates purinergic receptor expression and induces host-cell susceptibility to UTP-mediated apoptosis. Cell Microbiol (2011) 13:1410–28. 10.1111/j.1462-5822.2011.01630.x 21740498

